# Targeting Oncogenic Wnt/β-Catenin Signaling in Adrenocortical Carcinoma Disrupts ECM Expression and Impairs Tumor Growth

**DOI:** 10.3390/cancers15143559

**Published:** 2023-07-10

**Authors:** Morgan K. Penny, Antonio M. Lerario, Kaitlin J. Basham, Sahiti Chukkapalli, Dipika R. Mohan, Chris LaPensee, Kimber Converso-Baran, Mark J. Hoenerhoff, Laura Suárez-Fernández, Carmen González del Rey, Thomas J. Giordano, Ruolan Han, Erika A. Newman, Gary D. Hammer

**Affiliations:** 1 Doctoral Program in Cancer Biology, University of Michigan Medical School, Ann Arbor, MI 48109, USA; 2 Department of Internal Medicine, Division of Metabolism, Endocrinology and Diabetes, University of Michigan, Ann Arbor, MI 48109, USA; 3 Mott Solid Tumor Oncology Program, C.S. Mott Children’s and Women’s Hospital, Department of Surgery, University of Michigan, Ann Arbor, MI 48109, USA; 4 Medical Scientist Training Program, University of Michigan Medical School, Ann Arbor, MI 48109, USA; 5 UMH Frankel Cardiovascular Center Physiology and Phenotyping Core, Ann Arbor, MI 48109, USA; 6 In Vivo Animal Core, Unit for Laboratory Animal Medicine, University of Michigan Medical School, Ann Arbor, MI 48109, USA; 7 Department Head and Neck Oncology, Instituto de Investigación Sanitaria del Principado de Asturias, 33011 Oviedo, Spain; 8 Department of Pathology, Hospital Universitario Central de Asturias, Instituto de Investigación Sanitaria del Principado de Asturias, 33011 Oviedo, Spain; 9 Department of Pathology, University of Michigan Health System, Ann Arbor, MI 48109, USA; 10 Iterion Therapeutics, Inc., Houston, TX 77021, USA; 11 Endocrine Oncology Program, Rogel Cancer Center, University of Michigan Health System, Ann Arbor, MI 48109, USA

**Keywords:** adrenocortical carcinoma, adrenal, Wnt/β-catenin, Wnt, β-catenin, extracellular matrix, collagen, xenograft, targeted therapy, COL11A1

## Abstract

**Simple Summary:**

Adrenocortical carcinoma (ACC) is a rare, often deadly cancer arising from the adrenal gland. Mortality associated with ACC has remained unchanged over the last several decades. The rarity of ACC, an incomplete understanding of its molecular basis, and limited availability of pre-clinical models have hampered the development of novel therapeutic approaches. The present work aims to address these gaps with a focus on the Wnt/β-catenin cell signaling pathway, which is aberrantly activated in ~40% of ACC tumors. We discovered a novel ECM program activated in ACC that is associated with Wnt/β-catenin activation and poor survival. Wnt/β-catenin inhibition disrupted the expression of ECM genes and induced the loss of cancer cell viability. To extend these findings, we developed an orthotopic mouse model of rapidly progressive ACC and demonstrated that disruption of the Wnt/β-catenin axis with the novel small molecule inhibitor Tegavivint is a potential effective therapeutic strategy to reduce ACC tumor burden in vivo.

**Abstract:**

Adrenocortical carcinoma (ACC) is a rare but highly aggressive cancer with limited treatment options and poor survival for patients with advanced disease. An improved understanding of the transcriptional programs engaged in ACC will help direct rational, targeted therapies. Whereas activating mutations in Wnt/β-catenin signaling are frequently observed, the β-catenin-dependent transcriptional targets that promote tumor progression are poorly understood. To address this question, we analyzed ACC transcriptome data and identified a novel Wnt/β-catenin-associated signature in ACC enriched for the extracellular matrix (ECM) and predictive of poor survival. This suggested an oncogenic role for Wnt/β-catenin in regulating the ACC microenvironment. We further investigated the minor fibrillar collagen, collagen XI alpha 1 (COL11A1), and found that *COL11A1* expression originates specifically from cancer cells and is strongly correlated with both Wnt/β-catenin activation and poor patient survival. Inhibition of constitutively active Wnt/β-catenin signaling in the human ACC cell line, NCI-H295R, significantly reduced the expression of *COL11A1* and other ECM components and decreased cancer cell viability. To investigate the preclinical potential of Wnt/β-catenin inhibition in the adrenal microenvironment, we developed a minimally invasive orthotopic xenograft model of ACC and demonstrated that treatment with the newly developed Wnt/β-catenin:TBL1 inhibitor Tegavivint significantly reduced tumor growth. Together, our data support that the inhibition of aberrantly active Wnt/β-catenin disrupts transcriptional reprogramming of the microenvironment and reduces ACC growth and survival. Furthermore, this β-catenin-dependent oncogenic program can be therapeutically targeted with a newly developed Wnt/β-catenin inhibitor. These results show promise for the further clinical development of Wnt/β-catenin inhibitors in ACC and unveil a novel Wnt/β-catenin-regulated transcriptome.

## 1. Introduction

Adrenocortical carcinoma (ACC) is a rare cancer arising from the adrenal cortex. The overall 5-year survival rate for ACC is 35% [1], and 5-year survival is less than 10% for patients with stage 4 disease [2]. Although ACC patients have more favorable outcomes with surgical resection, the majority of patients either experience tumor recurrence following surgery or present with unresectable disease and require systemic strategies that routinely rely upon cytotoxic chemotherapies with limited benefit [2]. Significant efforts have been made to develop and implement multimodal therapy but provide limited benefit, with cisplatin, etoposide, doxorubicin plus mitotane (EDP-M) providing a median progression-free survival of only 5 months [3]. An improved understanding of ACC and stronger preclinical models are needed to develop rational therapies.

One molecular pathway that has been long associated with ACC is the Wnt/β-catenin pathway. This pathway is essential for development and homeostasis of many tissues, including the adrenal cortex [4,5]. Wnt ligand binding activates downstream signaling, leading to the cytoplasmic accumulation and nuclear translocation of β-catenin. In the nucleus, β-catenin serves as a transcriptional coactivator of TCF/LEF family transcription factors to upregulate target genes, including *AXIN2* [6], *LEF1* [7,8], and *APCDD1* [9]. In the absence of pathway activation, cytoplasmic β-catenin is targeted for degradation, but in human tumors, multiple genetic alterations can result in aberrant Wnt/β-catenin activation. These include loss-of-function (LOF) mutations in negative regulators *APC* and *ZNRF3* and gain-of-function (GOF) mutations in the gene encoding β-catenin, *CTNNB1*.

A connection between aberrant Wnt/β-catenin activation and ACC pathogenesis was first suggested by the increased incidence of adrenocortical tumors in patients with Familial Adenomatous Polyposis (FAP) [10]. FAP is a hereditary cancer syndrome characterized by a germline inactivating mutation in *APC*. Enhanced Wnt/β-catenin activation was further implicated in ACC by later studies that found *CTNNB1* GOF mutations and nucleo-cytoplasmic β-catenin accumulation in a relatively high proportion of human ACC cases [11]. More recently, large-scale genomic studies have confirmed that the genetic alterations that activate Wnt/β-catenin occur in nearly 40% of sporadic ACC cases [12,13]. These include GOF mutations in *CTNNB1* (~16%), LOF alterations in *APC* (2–3%), and LOF alterations in *ZNRF3* (~20%). Moreover, increased Wnt/β-catenin activity, determined by the presence of a genetic alteration or nucleo-cytoplasmic β-catenin staining, is associated with poor prognosis in ACC patients [14,15].

Given the high prevalence of genetic alterations that enhance Wnt/β-catenin signaling in ACC, there has been considerable interest in therapeutically targeting this pathway. Whereas upstream inhibitors that block Wnt ligand activation of cell surface receptors are attractive, activating mutations in *CTNNB1* are frequent in ACC, precluding the use of such inhibitors. Strategies that directly target downstream β-catenin activity are essential to effectively block pathway activation in these tumors. Whereas several previously developed small molecules have been shown to inhibit nuclear β-catenin binding to TCF/LEF [16], the clinical development of such therapies has not been successful. Tegavivint, a recently developed inhibitor currently in clinical trials, both disrupts the binding of β-catenin to transducin β-like protein 1 (TBL1, a key adaptor protein required for β-catenin binding and transcriptional activation) and promotes the SIAH-1-mediated degradation of nuclear β-catenin, which is not affected by *CTNNB1* GOF mutations at the phosphorylation site [17,18,19,20]. The on-target effects of Tegavivint demonstrating inhibition of Wnt/β-catenin transcriptional activity have been well documented in various in vitro and in vivo models of acute myeloid leukemia, multiple myeloma, and osteosarcoma [17,18,21,22] without overt toxicity at therapeutically effective doses [18,22].

Successfully advancing new therapeutic agents in the clinic requires in vivo models that faithfully recapitulate human disease. In ACC, the development of accurate preclinical xenograft mouse models has proven challenging. Most approaches rely on subcutaneous, renal subcapsular, or splenic injection of tumor cells [23,24,25,26]. These models require the injection of millions of cells (≥2.5 × 10^6^ cells) while failing to mimic the endogenous tumor microenvironment. Whereas genetic mouse mutants that spontaneously develop metastatic ACC have recently been described, the stochastic nature of tumor development limits the utility of such models in timely preclinical studies designed to test new therapeutic agents [27,28]. Orthotopic injection of Wnt/β-catenin-mutated ACC cells directly into the adrenal gland provides a more biologically relevant microenvironment to support ACC growth and allows for structured analyses and comparison of pharmacologically treated to non-treated tumors. This type of approach in other cancers has proven to more closely recapitulate the biology of human tumors with respect to vascularization, chemotherapy response, and metastasis while preserving the benefits of a xenograft model, including rapid and controlled tumor development capturing the heterogeneity of human cancer cells [29,30,31].

ECM proteins are key components of the tumor microenvironment that have been shown to play driving roles in both carcinogenesis and tumor progression [32,33]. Cell–matrix interactions can drive cell anchorage, spreading, and migration; growth factor interaction by sequestration; proliferation; and differentiation [34]. Abnormal ECM dynamics are a hallmark of cancer [32,35]. Although Wnt/β-catenin has been shown to be an essential paracrine signaling pathway mediating adrenocortical homeostasis [5,36] and the inhibition of β-catenin activity has been shown to inhibit the growth of ACC cells in culture [37,38,39,40], a role for Wnt/β-catenin regulating the expression of ECM components and the establishment of a tumor microenvironment has yet to be investigated.

In the present work, we identified a novel transcriptional signature of aberrant Wnt/β-catenin activation that is enriched in ECM components and associated with poor survival in ACC. We tested two independent inhibitors of Wnt/β-catenin, PKF115-584 and Tegavivint, and demonstrated that both effectively repressed Wnt/β-catenin activity, decreased the expression of a distinct subset of ECM genes, and coordinately inhibited the in vitro growth of ACC cells harboring a *CTNNB1* GOF mutation. To translate and extend our findings in vivo, we implemented and characterized a novel ultrasound-guided orthotopic xenograft model and found that Tegavivint treatment decreased β-catenin levels and significantly inhibited tumor growth in mice. Taken together, these studies support further clinical development of Wnt/β-catenin inhibitors that target β-catenin-dependent transcriptional activity in ACC.

## 2. Materials and Methods

### 2.1. Transcriptome Data Analysis

All analyses involving ACC-TCGA transcriptome data was performed in R (http://www.R-project.org/; accessed on 6 April 2021, version 4.1) using software packages from the Bioconductor portal (www.bioconductor.org; accessed on 6 April 2021, version 3.14). We downloaded harmonized RNA-seq counts data from the Genomic Data Commons (GDC) portal using *TCGABiolinks* (version 2.22.4) [41] and performed log2-cpm normalization using *EdgeR;* version 3.36.0 [42] after correcting for library size using the TMM method. We then used *MineICA* (https://rdrr.io/bioc/MineICA/; accessed on 6 April 2021, version 1.34.0) to perform Independent Component Analysis (ICA). We used the Mann–Whitney test to interrogate the association between each gene signature (component) identified by ICA and the presence of *CTNNB1* mutations by comparing the loading values of a “witness” gene (which is automatically determined by *MineICA*) in *CTNNB1-*mutated and *CTNNB1-*negative samples. We performed gene set enrichment analysis using the online *GSEA* tool (www.gsea-msigdb.org; accessed on 13 May 2020) to calculate enrichment scores using the Canonical Pathways collection from MSigDB, which is comprised of several curated gene lists from different sources, including Biocarta, KEGG, and Reactome, representing a broad collection of pathways and biologic processes. We used *ComplexHeatmap* (version 2.16.0) [43] to generate the heatmap representing the gene signature. We used the *GSVA* (Gene set variation analysis; version 1.42.0) default algorithm [44] to calculate the 5-gene canonical Wnt score as previously reported [28] and the score derived from the identified 340 genes. We used the Spearman test to calculate the correlations between selected ECM genes and the 5-gene Wnt score. We used the median 340-gene score to divide the cohort into two groups and estimate OS (overall survival) and DFS (disease-free survival) differences by Kaplan–Meier curves and the log-rank test. To divide the ACC-TCGA cohort into high- and low-expression groups according to *COL11A1* expression, we plotted the log2-CPM values of the gene as a function of the natural log-transformed hazard-ratio using smoothHR (https://github.com/arturstat/smoothHR; accessed on 6 April 2021, version 1.0.4). Using an additive Cox model, we defined the optimal cutoff as the point where the lower limit of the confidence interval of the natural log-transformed hazard ratio crossed the baseline. To perform the comparative analysis between the ACC-TCGA 340-gene Wnt signature identified by ICA and other models of constitutive Wnt activation, including the dataset by Leng et al. [36] and Heaton et al. [14], we downloaded the count table and .CEL files from GEO (accession numbers GSE144503 and GSE33371) and used *limma* (https://rdrr.io/bioc/limma; accessed on 6 April 2021, version 3.50.0) to perform class comparison analysis between β-Catenin-GOF and WT mice, and between nuclear CTNNB1+ and CTNNB1- human ACC. To compare the lists of genes upregulated by Wnt signaling in both human ACC and the murine model, we built a Venn diagram using the ggVennDiagram function (https://cran.r-project.org/web/packages/ggVennDiagram/index.html; accessed on 13 May 2020, version 1.2.2). To generate the chromatin accessibility track from ACC-TCGA samples, we downloaded 18 bigwig files from 9 ACC samples that were generated by Corces et al. [45] and combined them into a single bigwig file using BigWigMerge from UCSC tools (https://github.com/ucscGenomeBrowser/kent; accessed on 13 May 2020, version 357). To generate the track with the accessibility peaks, we combined the coordinates of the corresponding peaks reported by Corces et al. into a single .bed file using bedtools (https://bedtools.readthedocs.io/en/latest/index.html; accessed on 6 April 2021, version 2.30.0). To annotate these peaks, we downloaded .bed files of ChIP-seq experiments for TCF/LEF transcription factors in several different human cell lines from the UniBind database (https://unibind.uio.no; accessed on 6 April 2021). To demonstrate regions of open and active chromatin, as well as binding sites of CTNNB1 in the NCI-H295R cell line, we downloaded the bigwig files corresponding to ATAC-seq, H3K27ac, and CTNNB1 ChIP-seq from Mohan et al. [46], as well as corresponding bed files containing the peaks coordinates. We used the JBR browser (https://github.com/JetBrains-Research/jbr; accessed on 13 May 2020, version 1.0.5641) to generate the figures overlaying these tracks in the genomic regions of interest (Supplementary Figure S1).

### 2.2. Cell Culture and In Vitro Compounds

NCI-H295R (RRID:CVCL_0458) and Y1 cell lines were obtained from American Type Culture Collection (Manassas, VA, USA) and cultured in a humidified incubator containing 5% CO_2_ at 37 °C. NCI-H295R cells were grown in DMEM/Ham’s F-12 (1:1) (ThermoFisher Scientific, Waltham, MA, USA) supplemented with 10% NuSerum I (Corning, Corning, NY, USA), 1% Insulin-Transferrin-Selenium-Ethanolamine (ThermoFisher Scientific), and 1% penicillin/streptomycin (ThermoFisher Scientific). The human NCI-H2935R cell line has been authenticated using short tandem repeat profiling within the last three years. Y1 cells were cultured in High-Glucose DMEM (ThermoFisher Scientific) supplemented with 2.5% fetal bovine serum (Sigma-Aldrich, St. Louis, MO, USA), 7.5% horse serum (ThermoFisher Scientific), and 1% penicillin/streptomycin. All experiments were performed with mycoplasma-free cells. Tegavivint was a gift from Iterion Therapeutics, INC (Houston, TX, USA), and PKF115-584 was obtained from Tocris (Minneapolis, MN, USA). All compounds were solubilized in DMSO.

### 2.3. Assessment of Cell Viability

Cells were plated at a density of 300,000 cells per well in a clear 24-well plate or 30,000 cells per well in a 96-well plate one day prior to treatment. At the endpoint, cells were incubated with the colorimetric reagent alamarBlue (ThermoFisher Scientific) in accordance with the manufacturer’s instructions. Absorbance at 570 nm and 600 nm was measured after 3 h.

### 2.4. qRT–PCR

RNA was extracted using a RNeasy Mini Kit (QIAGEN, Hilden, Germany). Reverse transcription was performed using a High-Capacity cDNA Reverse Transcription Kit (Applied Biosystems, Waltham, MA, USA) according to the manufacturer’s instructions. The cDNA obtained was then used as the template for qPCR analysis using a Power SYBR Green PCR Master Mix (Applied Biosystems) and an Applied Biosystems 7300 Real-Time PCR System. Expression levels were normalized using the ΔΔCt method using *HPRT1* as a housekeeping gene. The primers used are listed in Supplementary Table S2.

### 2.5. Western Blots

Cells were harvested in RIPA buffer (ThermoFisher Scientific) containing cOmplete Protease Inhibitor Cocktail (Roche, Basel, Switzerland), PhosSTOP phosphatase inhibitors (Roche), and 50 uM ETDA, on ice for 30 min, and centrifuged at 16,000× *g* for 20 min at 4 °C. Protein concentrations were measured using a Pierce BCA protein assay kit (ThermoFisher Scientific), and equal amounts of total protein were separated by NuPAGE 4–12% Bis-TRIS gel in MES SDS running buffer (ThermoFisher Scientific). Protein was transferred from the gel to a nitrocellulose membrane and blocked with Odyssey Blocking Buffer (LiCor, Lincoln, NE, USA) for 1 h. Membranes were probed with antibodies against β-catenin (ThermoFisher Scientific MA1-2001, 1:1000), Active β-Catenin (Cell Signaling 9561 1:1500), or β-actin (Sigma-Aldrich A-5441, 1:5000) at 4 °C overnight. The next day, the membrane was washed three times with TBS-T buffer, incubated with the secondary antibodies for 1 h at room temperature, and washed three times with TBS-T and one time with PBS. Images were acquired using the LiCor Odyssey imaging system. Images were cropped to reorder samples run together on the same membrane.

### 2.6. Generation of Luciferase-Expressing Cell Lines

Stable NCI-H295R and Y1 cell lines expressing pLVX-EF1α-LUC2-IRES-mCherry [47], a gift from Dr. Judith Leopold (University of Michigan, Ann Arbor, MI, USA), were generated using lentiviral transduction and purified by FACS based on mCherry expression.

### 2.7. Animals and Animal Care

NSG mice were housed and maintained in specific pathogen-free conditions and facilities accredited by the American Association for Accreditation of Laboratory Animal Care and in accordance with current regulations and standards of the United States Department of Agriculture, United States Department of Health and Human Services. Animal care was overseen by the Unit for Laboratory Animal Medicine at the University of Michigan.

### 2.8. Xenograft Model of ACC

Intra-adrenal injections were performed on 6–13-week-old male immunodeficient NSG mice (The Jackson Laboratory, Bar Harbor, ME, USA) as described previously [48]. Ultrasound procedures and measurements were carried out using a Visual Sonics Vevo 2100 with a MS 550D 22–55 MHz or MS400 18–38 MHz transducer (University of Michigan Cardiovascular Center Research Core). Bioluminescent imaging was performed using an IVIS Spectrum in vivo imaging system (Perkin Elmer, Waltham, MA, USA) at the Center for Molecular Imaging, University of Michigan, using previously described methods [48]. For initial characterization studies, mice were euthanized after tumors exceeded 150 mm^3^, 10^11^ photons/s/cm^2^/sr, 120 days post-injection, ≥15% weight loss, or all other mice in the cohort had reached endpoint. For studies testing Tegavivint treatment, 200,000 NCI-H295R cells were injected. Treatment was initiated when the tumor volume reached approximately 40–100 mm^3^. Tumor volumes were extrapolated from last ultrasound using the NCI-H295R tumor doubling time of 6 days. Tegavivint was suspended in 5% dextrose, and an intraperitoneal (i.p.) injection of 50 mg/kg Tegavivint or vehicle was administered 5 days a week for two weeks. At necropsy, the tumor volume was calculated as an ellipsoid volume = 4/3 × π × (0.5 × D1) × (0.5 × D2) × (0.5 × D3), where D1, D2, D3 are the longest measurements in the X, Y, and Z axis, respectively.

### 2.9. Histopathology and Immunochemistry

Tissues were fixed in 10% neutral-buffered formalin for 24 h at room temperature, processed, paraffin embedded, and cut into 5 µm sections. Tissue characterization following routine hematoxylin and eosin staining was performed by a board-certified veterinary pathologist (MJH). For immunohistochemical staining, slides were labeled with antibodies against proCOL11A1 (DMTX1, supplied by Oncomatryx, S.L., Derio, Spain, 1:400, pH9), β-catenin (BD Biosciences 610153, 1:500, 10 mM citrate buffer pH6.3), SF-1 (Proteintech Group (PTGlabs) custom-made AB_2716716, 1:1000, 10mM NaCitrate + 0.05% Tween-20 pH6), Ki67 (ThermoFisher Scientific MA5-14520, 1:200, 10mM NaCitrate + 0.05% Tween-20 pH6), or Human Nucleoli (Abcam ab190710, 1:200, 10 mM citrate buffer pH6). Images were acquired on an Nikon Optiphot-2 microscope with an Olympus DP-70 camera. Scoring methodology: proCOL11A1 and β-catenin specimens were independently assessed in a blinded fashion by two observers following these criteria: proCOL11A1 immunostaining was evaluated according to the cytoplasmatic signal, scored on a 0–3 scale, with a panel of normal and non-malignant tissues used as negative controls, and pancreatic ductal adenocarcinoma used as a positive control. In total, 97 tumors, each with triplicate cores, were evaluated. For statistical analysis, tumors stained 0–2 were grouped. β-catenin immunostaining was evaluated as present or absent in the membranous, cytoplasmic, and nuclear compartments for each sample. For samples treated with Tegavivint, β-catenin nuclear signal was additionally evaluated in 4 HPF per sample and scored on a 0–3 scale. KI67 immunolabeling was quantified using the Aperio whole-slide imaging and digital analysis system. To quantify labeling, the nuclear algorithm provided was used following visual optimization and tuning to the labeling on KI67-stained slides.

For immunofluorescent staining, slides were blocked for 1 h at room temperature followed by primary antibody incubation overnight at 4 °C, and primary antibodies were detected with HRP-polymer solution (Vector Laboratories; Newark, CA, USA) and Alexa fluor tyramide reagent (Thermo Fisher; Waltham, MA, USA). Nuclei were counterstained with DAPI. Nonspecific staining was blocked using 2.5% horse serum for non-mouse antibodies. The M.O.M. kit (Vector Laboratories) was used for all primary mouse antibodies according to the manufacturer’s instructions. IF slides were mounted using ProLong Gold (Life Technologies, Carlsbad, CA, USA) and imaged on a Nikon Optiphot-2 microscope with a CoolSNAP Myo camera.

### 2.10. Statistics

Statistical analyses were performed using R (version 4.1), Graphpad Prism 7, or Microsoft Excel software (versions 16.71). *p*-values ≤ 0.05 were assigned significance, and data are expressed as mean ± SD. For comparison of Kaplan–Meier survival, a log-rank test was performed. For comparison between ACC cells or tumors treated with different experimental conditions, a two-tailed Student’s *t*-test or two-tailed Welch’s *t*-test (if normal distribution could not be assumed) was performed.

## 3. Results

### 3.1. A Wnt/β-Catenin-Driven Gene Signature Is Associated with Poor Patient Outcomes in ACC

**Figure 1:**
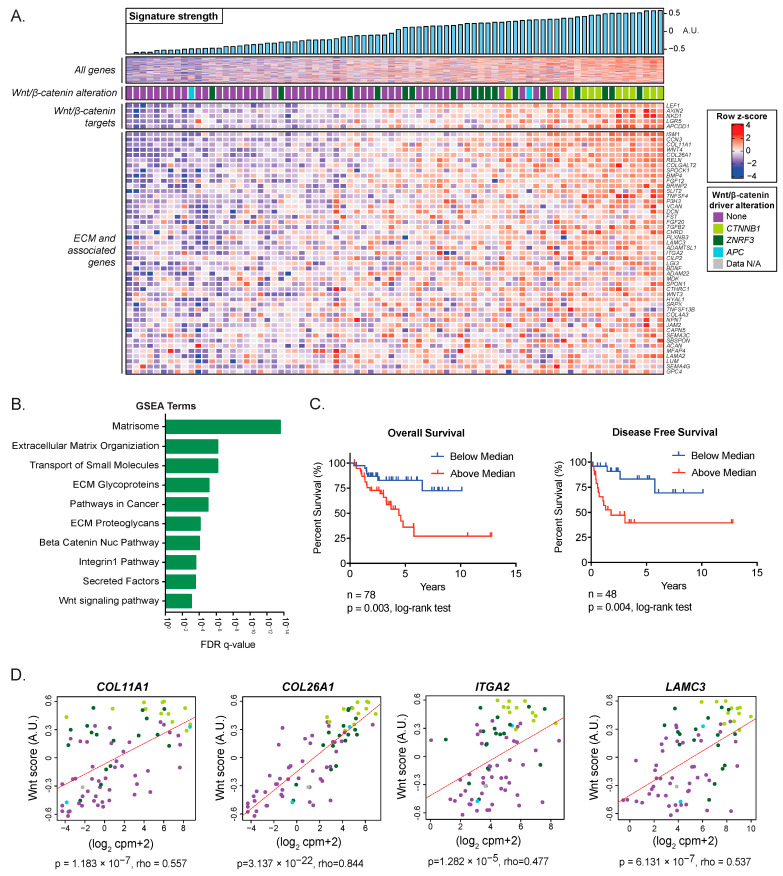
ACC-TCGA analysis identifies Wnt/β-catenin-associated ECM: (**A**). Independent component analysis of the TCGA transcriptome dataset in ACC identified a signature of coordinately expressed genes. A.U. stands for arbitrary units. (**B**). GSEA identified that the signature was enriched for the Wnt/β-catenin pathway and the expression of ECM and ECM-adhesion genes. (**C**). Kaplan–Meier analysis of the TCGA cohort indicated that patients with component signature expression above the median show shorter overall survival (OS) (high score n = 39, low score n = 39). An expression higher (n = 24) or lower (n = 24) than the median also predicted shorter disease-free survival (DFS). (**D**). The rxpression of *COL11A1*, *COL26A1*, *ITGA2*, and *LAMC3* was significantly correlated with a Wnt score representing the expression of bona fide Wnt/β-catenin target genes *AXIN2*, *LEF1*, *APCDD1*, *NKD1*, and *LGR5*.

To identify transcriptional programs that are engaged in human ACC tumors, we analyzed RNA-seq data from 78 ACC samples included in The Cancer Genome Atlas Project (ACC-TCGA) [12]. Using unsupervised ICA, we identified a signature of 340 coordinately expressed genes (Supplementary Table S1) that was significantly increased in tumors with somatic activating mutations in *CTNNB1* (*p* = 3.84 × 10^−7^, Figure 1A). This gene signature included a spectrum of Wnt/β-catenin target genes, including *AXIN2, LEF1, NKD1, APCDD1,* and *LGR5* (Figure 1A). To investigate the biologic processes enriched in this signature, we performed gene set enrichment analysis (GSEA) using the MSigDB Canonical Pathways gene set, which is comprised of several curated gene lists representing a broad collection of pathways and biologic processes. Consistent with the high prevalence of *CTNNB1* mutations, nuclear β-catenin signaling was significantly enriched in this component (Figure 1B, FDR q-value = 1.03 × 10^−4^).

Given the previously reported associations between Wnt/β-catenin pathway activation and poor outcomes in ACC [14,15], we sought to determine whether this association was also captured by the identified 340-gene signature. We used *GSVA* to calculate a score that quantified the expression of these 340 genes in each sample. We divided the ACC-TCGA cohort into two groups according to the median of the gene score. We observed a significant difference in both overall survival and disease-free survival between the two groups (OS *p* = 0.003; DFS *p* = 0.004) (Figure 1C). These results support that aberrant Wnt/β-catenin activation is associated with poor patient outcomes in ACC, consistent with prior studies [14,15].

### 3.2. Wnt/β-Catenin Activity Is Associated with ECM Expression in ACC

In addition to these findings, GSEA revealed that the Wnt/β-catenin-associated signature was most heavily enriched for ECM, ECM-receptor proteins, and other ECM-associated proteins, including collagens, integrins, laminins, and secreted factors (Figure 1B). Given the strong association between this gene signature and *CTNNB1* mutation, we hypothesized that the signature, in which ECM biology is among the most significantly represented processes, was Wnt/β-catenin-driven. To further investigate this hypothesis, we performed a correlation analysis of select ECM genes and known Wnt/β-catenin target genes. We analyzed the ECM genes that provided the highest component projections in the signature identified by ICA in the classes of collagen, laminin, and integrin: *COL11A1*, *COL26A1*, *LAMC3*, and *ITGA2* (Supplementary Table S1). To more accurately measure Wnt/β-catenin pathway activation and not rely on a single gene readout, we calculated a Wnt score based on the combined expression of five bona fide Wnt/β-catenin target genes [28]. Our analysis showed a significant positive correlation between the Wnt score and each of the ECM genes (Figure 1D). Notably, tumors with *CTNNB1* mutations exhibited the highest Wnt scores and the highest expression of ECM genes. To validate these findings and to better understand the context in which constitutive Wnt signaling deregulates ECM genes, we performed a comparative analysis using another human adrenocortical tumor dataset annotated with the nuclear CTNNB1 status of each sample [14] and a mouse model of adrenal-specific β-Catenin GOF [36]. Whereas bona fide canonical Wnt targets were upregulated in both ACC datasets and the murine model, the Wnt ECM signature was specific to human ACC, suggesting a cancer-specific regulatory mechanism (Supplementary Figure S2A,D–F). Additional analysis using publicly available datasets [45], including ChIP-seq data generated in the NCI-H295R cell line [46], demonstrated β-catenin and TCF/LEF binding in the promoter and/or putative distal regulatory elements of our selected subset of ECM genes, overlapping with regions of active and accessible chromatin (Supplementary Figure S1), suggesting β-catenin dependent regulation. The complete results of these analyses, including the lists of differentially expressed genes and overlaps in the Venn diagram, are included in Supplementary Table S1.

**Figure 2:**
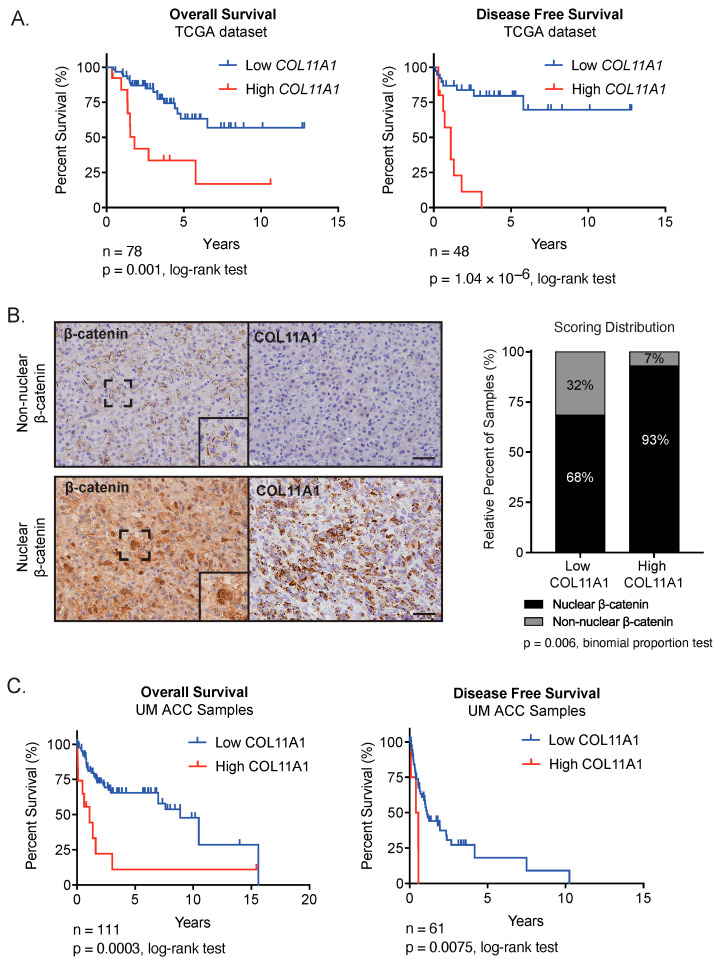
COL11A1 expression is correlated with Wnt/β-catenin activation in human ACC samples and predicts outcome: (**A**). Correlation of OS and DFS with *COL11A1* expression by Kaplan–Meier analysis in TCGA datasets showed that high *COL11A1* transcript expression was correlated with decreased OS (n = 66 low, n = 12 high, log-rank test *p* = 0.001) and DFS (n = 38 low, n = 10 high, log-rank test *p* = 1.04 × 10^−6^). (**B**). Serial sections from patient ACC samples (n = 97) collected at the University of Michigan and stained for β-catenin and COL11A1 showed that COL11A1 expression was correlated with nuclear β-catenin localization (binomial proportions test *p* = 0.006). Representative serial sections shown from a tumor with non-nuclear β-catenin localization versus nuclear β-catenin localization. Scalebar, 100 µM. (**C**). Kaplan–Meier analysis of OS and DFS following COL11A1 staining of University of Michigan ACC samples showed that high COL11A1 staining was correlated with shorter OS (n = 98 low, n = 13 high, log-rank test *p* = 0.0003) and shorter DFS (n = 56 low, n = 5 high, log-rank test *p* = 0.0075).

High expression of minor fibrillar collagen *COL11A1* has been associated with disease progression and poor survival in ovarian and other cancers [49]. We therefore chose to investigate the expression of this gene further. We analyzed patient data from ACC-TCGA and found that high *COL11A1* transcript expression was associated with significantly shorter OS and DFS (Figure 2A, Supplementary Figure S2B,C). To follow up on these findings, we performed immunohistochemistry on tissue microarrays (TMAs) containing 97 ACC samples. Recent work has determined that ACC has one of the lowest contributions of stromal cells across cancer types [12,50], suggesting that ECM may be at least in part tumor-cell-derived. In the current study, we observed COL11A1 expression in the ACC tumor parenchyma. Moreover, we observed that COL11A1 staining was significantly enriched in tumors with nuclear β-catenin localization (*p* = 0.006) (Figure 2B), indicating that the expression of COL11A1 protein is associated with oncogenic Wnt/β-catenin activation. We observed that COL11A1 protein expression was also significantly associated with shorter OS (*p* = 0.0003) and DFS (*p* = 0.0075) (Figure 2C).

### 3.3. Inhibition of Wnt/β-Catenin Reduces ACC Viability and Disrupts ECM Expression

**Figure 3:**
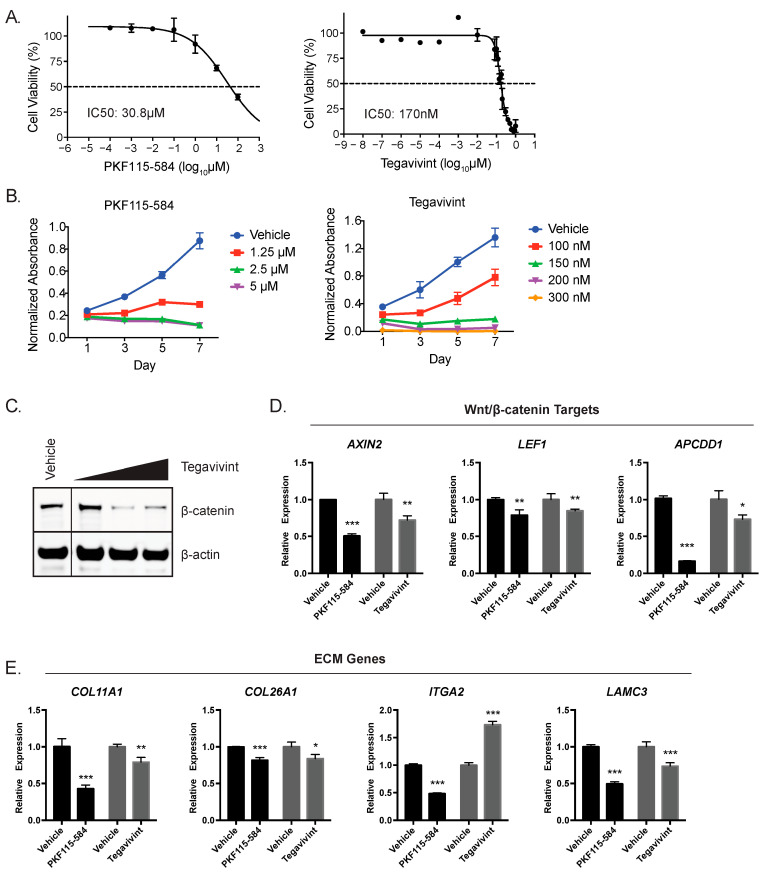
Constitutively Wnt/β-catenin-active ACC cells are sensitive to β-catenin inhibition in vitro: (**A**). IC50 for NCI-H295R ACC cells treated with PKF115-584 or Tegavivint for 24 h. (**B**). β-catenin inhibition with PKF115-584 or Tegavivint treatment led to significantly decreased NCI-H295R viability, suggesting that Wnt/β-catenin signaling may regulate cell viability in ACC. (**C**). Representative western blot following 24 h treatment with 100 nM, 150 nM, or 200 nM Tegavivint. Images were cropped to reorder samples run together on the same membrane. (**D**). Gene expression of Wnt/β-catenin targets in NCI-H295R cells following 24 h treatment with 100 nM Tegavivint or 1.25 µM PKF115-584. (**E**). Gene expression of Wnt/β-catenin-associated ECM in NCI-H295R cells, following 24 h treatment with 100 nM Tegavivint or 1.25 µM PKF115-584. n  ≥  3 biologic replicants for all experiments; *p*-value was calculated using a two-tailed Student’s *t*-test. Data are presented as mean  ±  SD; * *p* < 0.05; ** *p* < 0.005, *** *p* < 0.0005. Original western blots presented in File S1.

Having established that Wnt/β-catenin activity and *COL11A1* expression were correlated with poor outcomes in patients, we next wanted to evaluate the effects of Wnt/β-catenin inhibition on the ECM gene signature and investigate the utility of inhibition as a therapeutic strategy in ACC. We performed experiments on the NCI-H295R human ACC cell line, which harbors an activating S45P *CTNNB1* mutation [11] and demonstrates high levels of Wnt/β-catenin activity compared to Y1 ACC cells (Supplementary Figure S3A). NCI-H295R cells were treated with Tegavivint, a recently developed Wnt/β-catenin inhibitor in clinical development [17,21], or PKF115-584, a well-established Wnt/β-catenin inhibitor [16] previously shown to inhibit cell growth in vitro in ACC [37]. Both inhibitors caused a dose-dependent inhibition of cell growth and viability (Figure 3A,B), with NCI-H295R cells showing greater sensitivity than Y1 cells (Supplementary Figure S3B,C). Given that Tegavivint causes a decrease in β-catenin levels [17,18], we investigated the effect of treatment in NCI-H295R cells and found that Tegavivint resulted in a robust decrease of GOF β-catenin protein (Figure 3C).

To specifically interrogate the link between Wnt/β-catenin transcriptional activity and cell autonomous ECM expression in ACC cells, we treated NCI-H295R cells with Tegavivint and PKF115-584 and measured gene expression changes by qRT-PCR. These two pharmacologic inhibitors diverge in mechanisms of β-catenin inhibition—Tegavivint disrupts β-catenin TBL1/SIAH-1 interaction [17,18,19,20], and PKF115-584 disrupts β-catenin binding to TCF [37]. Both inhibitors effectively blocked β-catenin-mediated transcriptional activation, as evidenced by the significantly reduced expression of *AXIN2*, *LEF1*, and *APCDD1* (Figure 3D). Moreover, Wnt/β-catenin inhibition significantly decreased *COL11A1*, *COL26A1*, and *LAMC3* expression (Figure 3E). Pathway inhibition had variable effects on the expression of *ITGA2*, suggesting variable mechanisms of β-catenin-dependent regulation of gene expression compared to the other genes. These results demonstrate that the pharmacological disruption of Wnt/β-catenin activity in ACC also decreases the expression of ECM components.

### 3.4. An Orthotopic Xenograft Model of ACC Recapitulates High-Grade ACC with Metastatic Potential

We next wanted to test the efficacy of Wnt/β-catenin inhibition in vivo. Preclinical models of ACC are limited, and existing heterotopic xenografts, including subcutaneous models, fail to mimic the endogenous tumor microenvironment [23,25,26]. The development and use of orthotopic xenografts, however, has been limited by complex and morbid murine surgery [27,34]. To overcome these limitations, we established and characterized a novel orthotopic ACC xenograft model utilizing the ultrasound-guided implantation of tumor cells [48].

**Figure 4:**
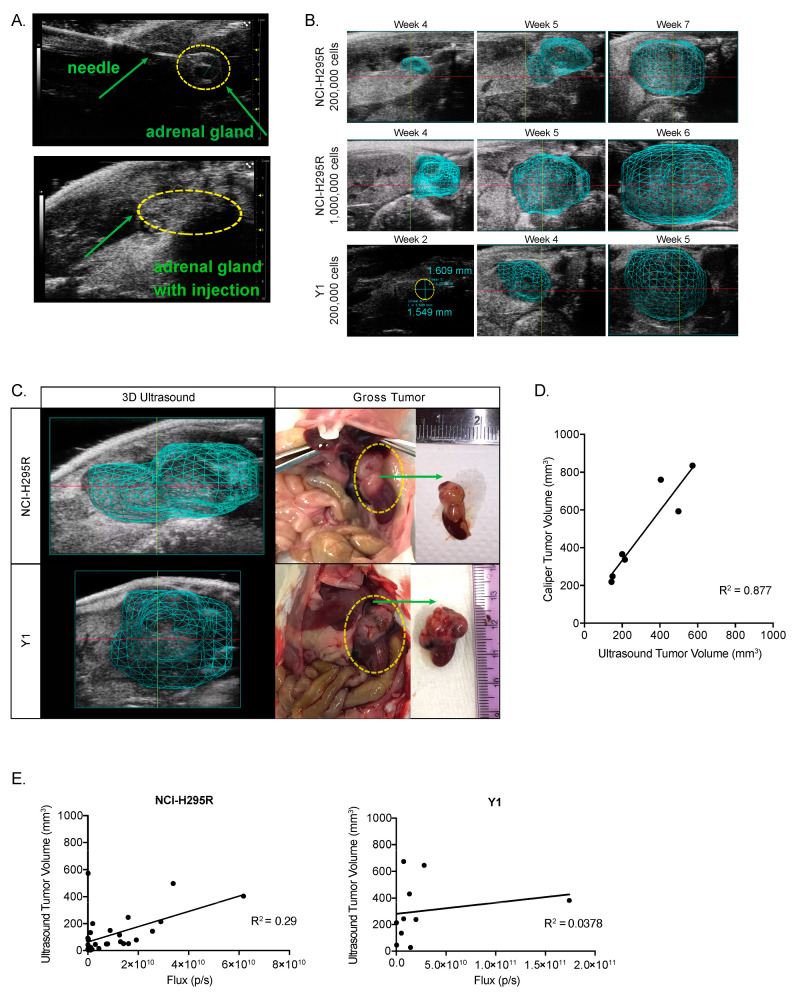
Xenograft implantation and tumor progression: (**A**). In vivo injection process (**B**). Adrenals and the growth of tumors were monitored using ultrasound, with a 3D reconstruction of the tumor boundaries when a tumor was detected (**C**). Here, tumor boundaries mapped from ultrasound prior to necropsy (left panels). Dashed yellow circles in middle panels show macroscopic appearance of the tumors before resection. Left panels: macroscopic gross appearance of the tumors after resection. (**D**). Ultrasound tumor volume and tumor volume calculated from caliper measurements are strongly correlated (linear regression R^2^ = 0.877). (**E**). In vivo bioluminescent signal is plotted against ultrasound tumor volume (NCI-H295R linear regression R^2^ = 0.29; Y1 linear regression R^2^ = 0.0378).

First, we generated two stable ACC cell lines, NCI-H295R and Y1 cells, expressing a luciferase construct for in vivo visualization [47]. We then used ultrasound guidance to inject 200,000 Y1 or NCI-H295R cells in the left adrenal gland of NSG immunocompromised mice (Figure 4A). An additional group of animals was injected with 1,000,000 NCI-H295R cells since NCI-H295R xenografts have previously required millions of cells to achieve efficient tumor engraftment [23]. Following implantation, we monitored mice for tumor growth. Because tumor growth in the retroperitoneal space cannot be measured directly, we used ultrasound with 3D reconstruction of tumor boundaries as well as bioluminescent imaging to characterize tumor engraftment and progression (Figure 4B). All groups demonstrated ≥80% engraftment efficiency (Table 1).

To validate the accuracy of ultrasound tumor volume reconstruction, we compared ultrasound measurements taken just prior to necropsy with the gross tumor specimen. We found that 3D ultrasound tumor volume was a sensitive and accurate measurement of tumor morphology (Figure 4C) that strongly correlated with the tumor volume calculated from caliper measurements at necropsy (R^2^ = 0.877) (Figure 4D). We also compared the bioluminescent signal to the ultrasound volume and found that the total bioluminescent flux (photons/second) measured in vivo did not correlate linearly with the tumor volume (Figure 4E). Taken together, these data support 3D ultrasound as a more accurate and preferred method for adrenal tumor monitoring.

**Figure 5:**
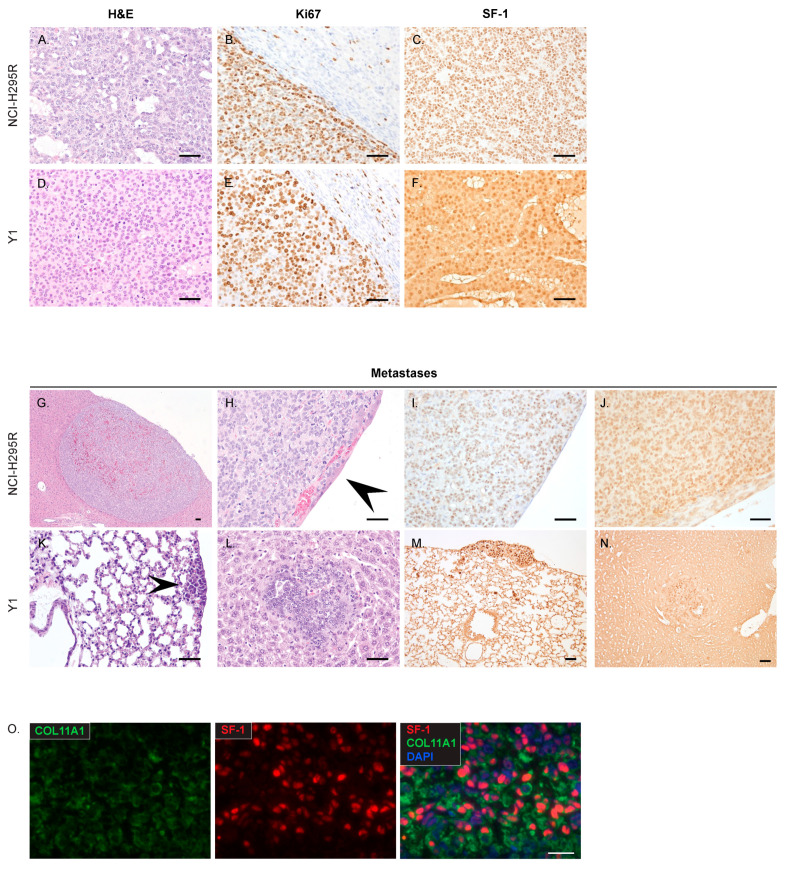
Histology of primary tumors and metastatic growths: (**A**). NCI-H295R tumors were characterized as composed of an expansile and focally infiltrative proliferation of packets, clusters, and sheets of neoplastic epithelial cells interspersed with variably sized cystic spaces. (**B**). Ki67 staining of an NCI-H295R tumor invading the adjacent kidney (**C**). Representative SF-1 staining of NCI-H295R tumors. (**D**). Tumors formed from Y1 cells were composed of ribbons, clusters, and lobules of poorly differentiated epithelial cells separated by a fine fibrovascular stroma, with multifocal areas of hemorrhage and necrosis. (**E**). Y1 Ki67 staining of tumor and adjacent kidney. (**F**). Representative SF-1 staining of a Y1 tumor. (**G**,**H**). NCI-H295R liver metastasis, overlain with a layer of hepatocytes. (**I**). Anti-human Nucleolar staining of NCI-H295R metastasis. (**J**). SF-1 staining of NCI-H295R metastasis. (**K**). Y1 lung metastasis. (**L**). Y1 liver metastasis. (**M**,**N**). SF-1 staining of Y1 metastases. (**O**). NCI-H295R xenograft tumor with COL11A1 and SF-1 immunofluorescent staining demonstrates co-expression in ACC cells. Scalebar, 100 µM. Arrowheads indicate metastatic lesions in panels H and K.

At necropsy, tumors were further characterized to identify the utility of the model for future studies. Histologically, tumors formed from NCI-H295R and Y1 xenografts modeled characteristics of high-grade ACC, designated by 20 or greater mitotic figures per 50 high-power fields (≥20/50 HPF) (Figure 5A,D). NCI-H295R tumors formed following injection with 200,000 cells and 1,000,000 cells had >20/50 HPF (mean 582.5 ± 79.3 SD; mean 570 ± 133.3 SD) and a high Ki67 labeling index (Figure 5B). All Y1 tumors had >20/50 HPF (mean 346.7 ± 228.1 SD) and a high Ki67 labeling index (Figure 5E). Tumors and metastasis also retained the expression of adrenocortical marker steroidogenic factor-1 (SF-1) (Figure 5C,F). Importantly, xenografted tumors formed distant metastases in the liver and lung, sites characteristic of ACC (Table 1), NCI-H295R metastases exhibited anti-human nucleolar staining, consistent with its human origin (Figure 5I), and all the metastatic lesions retained SF-1 expression (Figure 5J,M,N). Whereas NCI-H295R metastases were found in the liver in one mouse with long tumor latency (Figure 5G,H; Table 1), Y1 metastases frequently formed in the liver and lung (Figure 5K,L; Table 1). Our characterization establishes that orthotopic adrenal xenografts model high-grade, advanced ACC. In addition, the staining of NCI-H295R tumors found co-expression of the steroidogenic marker SF-1 and COL11A1, further demonstrating that COL11A1 is expressed by ACC cells (Figure 5O).

### 3.5. Tegavivint Inhibits High-Grade ACC Growth In Vivo

**Figure 6:**
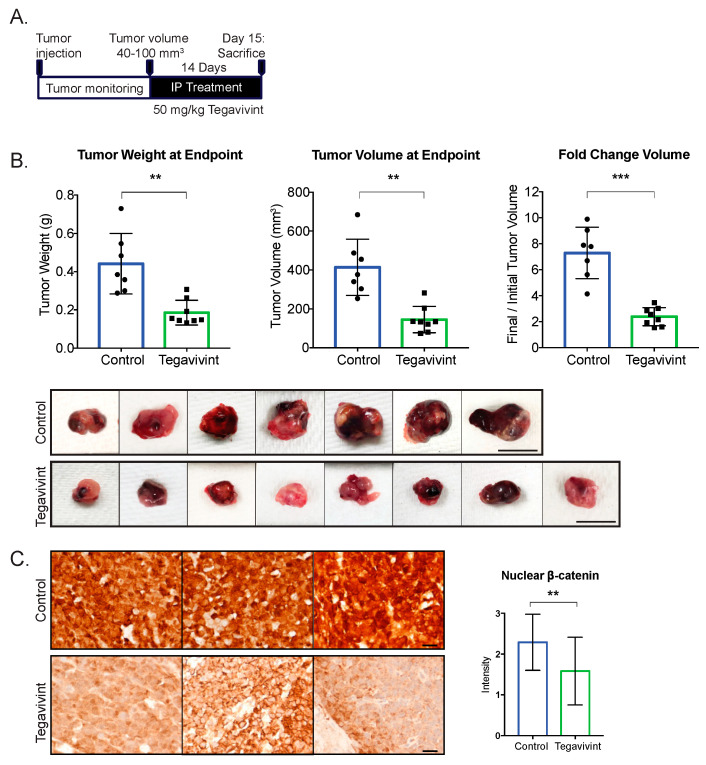
β-catenin inhibition significantly reduces tumor growth: (**A**). Experimental scheme of study design. NSG mice were injected with 200,000 NCI-H295R cells in the left adrenal. Treatment was started when xenografts reached 40–100 mm^3^. Mice were treated with 50 mg/kg Tegavivint or vehicle 5 days per week (Monday–Friday) for 14 days. (**B**). Tegavivint treatment significantly decreased tumor weight and volume at endpoint. *p*-value was calculated using two-tailed Welch’s t-test. Data are presented as mean  ±  SD; ** *p* < 0.005, *** *p* < 0.0005. Scalebar, 10 mm. (**C**). Murine ACC tumors treated with 50 mg/kg Tegavivint and stained for β-catenin. Scalebar, 100 µM. Nuclear β-catenin staining was quantified by two independent observers and was significantly decreased in Tegavivint-treated tumors.

We next evaluated the effect of β-catenin inhibition on tumor growth in our xenograft model. To best model therapeutic treatment in patients with existing ACCs, we implanted NCI-H295R cells, which harbor an activating GOF β-catenin mutation. Tumors were allowed to grow to 40–100 mm^3^, after which they were treated with 50 mg/kg Tegavivint (n = 8), or vehicle (n = 7) (Figure 6A). There was no significant difference in estimated tumor volume at the onset of treatment (*p* = 0.936), and Tegavivint was well tolerated by mice. After two weeks of Tegavivint treatment, a striking 65% reduction in tumor weight and 58% reduction in tumor volume was observed over vehicle-treated controls (*p* = 0.0047, *p* = 0.0015, respectively; Figure 6B). Tumors contained extensive areas of necrosis (Supplementary Figure S4A), preventing an accurate capture of gene or protein expression; however, the staining of tumors at an earlier timepoint showed that Tegavivint produced a reduction in β-catenin levels when compared to robust nuclear and cytoplasmic expression in the tumors of vehicle-treated mice (Figure 6C). At this early timepoint, we were unable to capture differential downstream changes in the protein expression of LEF1 (Supplementary Figure S4B). Taken together, the data indicate that Wnt/β-catenin inhibition is a potentially efficacious therapeutic strategy for high-grade ACC harboring activating mutations in the pathway.

## 4. Discussion

Over the last two decades, a large body of evidence has implicated sustained Wnt/β-catenin signaling as a key driver event in the molecular pathogenesis of ACC [51]. Indeed, we now know that somatic alterations targeting Wnt/β-catenin pathway prevail in nearly 40% of ACC [12,13]. Furthermore, genetic mouse models bearing adrenal-specific activation of Wnt/β-catenin exhibit adrenocortical hyperplasia, dysplasia, and tumor formation [14,28,52]. Whereas these studies strongly support a dominant role for Wnt/β-catenin in the molecular pathogenesis of ACC, much is unknown about the downstream mechanisms by which Wnt/β-catenin promotes tumor growth. Using ICA, we characterized a strong effect of Wnt/β-catenin activity on the ACC transcriptome. Our data build on previous studies [14,38] to identify a 340-gene signature expressed in ACCs with *CTNNB1* mutations that is highly correlated with both Wnt/β-catenin activity and uniquely associated with ECM expression. The strong transcriptional signature associated with the activation of this pathway and its major contribution to decreased patient survival suggest that pharmacologically targeting Wnt/β-catenin is an attractive therapeutic approach that can fulfill the urgent need to improve the standard of care for advanced ACC [2,3]. Indeed, we showed here that the transcriptional and tumor viability programs coordinated by Wnt/β-catenin in ACC are sensitive to the pharmacological inhibition of this pathway.

Our analysis sought to better understand the role of Wnt/β-catenin signaling in ACC. Interestingly, we observed a spectrum of Wnt/β-catenin signaling across ACC, with patient tumors bearing ligand-independent GOF mutations in β-catenin exhibiting the highest level of downstream Wnt signaling, whereas tumors with ligand-dependent LOF mutations in ZNRF3 exhibited more moderate Wnt/β-catenin activation, as previously observed [28]. ACCs with ligand-dependent signaling demonstrated a spectrum of Wnt target gene expression. Furthermore, we observed that the expression of *COL11A1*, *COL26A1*, *LAMC3*, and *ITGA2* was correlated with Wnt/β-catenin pathway activation (Figure 1D). Though our analysis of TCGA data is limited by the absence of normal adrenal tissue, we hypothesize that only highly active Wnt/β-catenin signaling can induce expression of select ECM genes in ACC, driving a cancer-specific tumor microenvironment. This is further supported by a previous study from our group, which did not identify ECM genes as a signature of Wnt-responsive cells in the adrenal cortex [53].

Though mutation data are not available for the TMA tumors we characterized, we assume that nuclear β-catenin staining captures tumors with a spectrum of Wnt/β-catenin activity, including those with either *CTNNB1* and *ZNRF3* mutations. This is likely to be the case as the observation that a significant proportion of ACC bear nuclear β-catenin staining not explained by *CTNNB1* mutation status preceded the discovery of *ZNRF3* mutations in ACC [15]. Our characterization found that nearly all tumors exhibiting COL11A1 protein expression had nuclear β-catenin. Such an observation, together with the decrease in the expression of a subset of ECM genes following the inhibition of Wnt/β-catenin signaling, is consistent with nuclear or active β-catenin being necessary to induce cell autonomous COL11A1 expression. Although it is unclear what other factors may influence this relationship, the strength of Wnt/β-catenin signaling is predicted to be a critical factor in determining the expression of COL11A1 in ACC, and IHC is likely sensitive to capture lower levels of Wnt/β-catenin signaling insufficient to drive COL11A1 expression.

We speculate that the correlation between Wnt/β-catenin and ECM is more broadly applicable. Indeed, differential ECM secretion has been linked to Wnt/β-catenin signaling in Ewing Sarcoma cells [54]. Whereas our results support that the Wnt/β-catenin-regulated transcriptome in ACC is heavily enriched for ECM and demonstrate that COL11A1 accumulates in ACC samples with nuclear β-catenin staining, it is predicted that additional signaling pathways can influence ECM gene expression as well. We hypothesize that this is likely the case for *ITGA2*, where we observed a variable response to Wnt/β-catenin inhibitors. Indeed, the regulation of ITGA2 expression has been previously linked to not only β-catenin but also the transcription factor FOXL2, as well as AP1, AP2, and GATA [55].

In other studies, COL11A1 expression has been almost exclusively localized to cancer-associated stromal cells [49,56]. In contrast, ACC is one of the most stroma-poor cancer types in the TCGA cohort [12], and we found that *COL11A1* expression is present in both NCI-H295R cells and in the neoplastic cells present in human ACC samples. Moreover, TCGA data confirm that more aggressive subtypes of ACC are both enriched for Wnt/β-catenin and exhibit the lowest level of stromal cell infiltration, consistent with a cell autonomous role of Wnt/β-catenin activity in ECM deposition [12,50]. Though stromal contribution to ECM cannot be ruled out, our studies support that ACC cells provide a biologic contribution to the tumor microenvironment.

Our finding that *COL11A1* transcript and protein expression is associated with shortened OS and DFS is consistent with findings in ovarian cancer [57] and complementary to findings that elevated *COL11A1* expression is associated with chemoresistance in human tumors [58]. These results pose the intriguing question of whether ECM and COL11A1 specifically promote ACC aggressiveness and progression.

Given our desire to ultimately target therapy against the Wnt/β-catenin pathway, we developed a model to translate ACC in vitro findings in vivo. We implemented and characterized a novel model of ACC that captures features of high-grade ACC in patients. The high mitotic rate and Ki67 labeling index of both NCI-H295R and Y1 tumors capture histologic features of patient tumors with high risk for recurrence and decreased overall survival [2,59]. We also showed that orthotopic adrenal implantation can lead to spontaneous metastatic growth in the liver and the lungs, which are the most frequent sites of ACC metastasis in patients [1]. Therefore, this study represents the first reported model of high-grade, spontaneously metastatic ACC using currently available cell lines. Though we did not compare orthotopic xenografts to subcutaneous or other xenografts in a controlled study, orthotopic implantation allows for modeling the microenvironment within the anatomic location of primary tumor initiation. Traditionally, intra-adrenal implantation has lacked implementation for preclinical therapeutic investigations because of the extensive surgical procedure and post-surgical monitoring [60]. In contrast, our approach employing minimally invasive, ultrasound-guided implantation enabled high-throughput injection procedures, efficient tumor engraftment, and rapid recovery. We believe that the methodologies presented here will enable a broader implementation of orthotopic xenografts in ACC research.

We used our preclinical tumor model to extend our in vitro findings and confirm that the therapeutic inhibition of Wnt/β-catenin significantly inhibits the growth of tumors harboring a GOF *CTNNB1* mutation. Our results are consistent with and complimentary to prior studies in which β-catenin knockdown reduced Wnt/β-catenin transcriptional activity in ACC cells and impaired the function of β-catenin in cell-cell adhesion [36,39]. Taken together, these data demonstrate preclinical support for further study on the clinical utility of Wnt/β-catenin inhibitors in ACC. Currently, Tegavivint is undergoing clinical testing (NCT04780568, NCT04874480, NCT04851119) in a variety of Wnt/β-catenin active cancers. It will be important to test the effectiveness of Wnt/β-catenin inhibitors in ACC as single agents or in combination with other therapeutic modalities. Our model complements findings from a recently developed genetic mouse model of β-catenin GOF with p53 loss of function, which represents an alternate approach for testing further therapies directed against Wnt/β-catenin [27].

In conclusion, we identified that ACC with high Wnt/β-catenin activity exhibits a unique expression pattern of ECM components associated with poor survival. Moreover, Wnt/β-catenin inhibition disrupts ECM expression and reduces growth in ACC with an activating *CTNNB1* mutation. Importantly, we developed a novel orthotopic xenograft model to investigate the preclinical implications of these findings and found that the in vivo inhibition of Wnt/β-catenin:TBL1 signaling with Tegavivint significantly reduced tumor growth. Collectively, our studies demonstrate the preclinical efficacy of a Wnt/β-catenin inhibitor in ACC and underscore the rationale for therapeutically translating Wnt/β-catenin inhibition in ACC patients.

## Figures and Tables

**Table 1:** Tumor characteristics following orthotopic xenograft implantation.

Cell Line	Cell Number	Average Time to Detection (SD)	Average Time to Endpoint (SD)	Tumor Engrafment	Liver Metastasis	Lung Metastasis
Y1	2 × 10^5^	2.5 weeks (0.9)	5.1 weeks (1.0)	89%	50%	83%
NCI-H295R	2 × 10^5^	3.6 weeks (0.9)	8.9 weeks (3.0)	80%	0%	0%
	1 × 10^6^	3.4 weeks (0.7)	6.9 weeks (2.7)	100%	13%	0%

## Data Availability

Data sources and handling of the publicly available datasets used in this study are described in the Materials and Methods. Further details and other data that support the findings of this study are available from the corresponding authors upon request.

## Supplementary Materials

The following supporting information can be downloaded at: https://www.mdpi.com/article/10.3390/cancers15143559/s1, Figure S1: Epigenetic data support a direct regulation of extracellular matrix genes by β-catenin.; Figure S2: β-catenin-driven extracellular matrix signature is cancer-specific and associated with poor outcomes.; Figure S3: Pharmacological inhibition of β-catenin induces on-target loss of viability *in vitro*.; Figure S4: Central necrosis and variable decrease in β-catenin in tumors following two weeks of Tegavivint treatment; Table S1: Component genes with annotations; Table S2: Oligonucleotides used in this study. File S1: Original western blots.

## Abbreviations

ACC	Adrenocortical carcinoma
DFS	Disease-free survival
ECM	Extracellular matrix
FAP	Familial Adenomatous Polyposis
GSEA	Gene set enrichment analysis
GOF	Gain-of-function
GSVA	Gene set variation analysis
HPF	High-power fields
ICA	Independent Component Analysis
OS	Overall Survival
LOF	Loss-of-function
SD	Standard Deviation
TCGA	The Cancer Genome Atlas Project
TMA	Tissue Microarray
