# Supplementary material for: Targeting Oncogenic Wnt/β-Catenin Signaling in Adrenocortical Carcinoma Disrupts ECM Expression and Impairs Tumor Growth
Source: Cancers (Basel). 2023 Jul 10;15(14):3559. doi: 10.3390/cancers15143559 (PMC10377252; doi:10.3390/cancers15143559)
Supplement: Supplementary file 1 [file cancers-15-03559-s001.zip › Supplementary Table S1a - Component genes with annotations copy.pdf]

**Supplementary Table S1 - Component genes with annotations**

| Gene_Symbol | Description | Scaled_projection |
|---|---|---|
| ISM1 | isthmin 1 [Source:HGNC Symbol;Acc:HGNC:16213] | 10.25453533 |
| COL11A1 | collagen type XI alpha 1 chain [Source:HGNC Symbol;Acc:HGNC:2186] | 9.802373369 |
| CCN3 | cellular communication network factor 3 [Source:HGNC Symbol;Acc:HGNC:7885] | 9.716146941 |
| CALN1 | calneuron 1 [Source:HGNC Symbol;Acc:HGNC:13248] | 9.403110747 |
| LGR5 | leucine rich repeat containing G protein-coupled receptor 5 [Source:HGNC Symbol;Acc:HGNC:4504] | 9.303881267 |
| PCP4 | Purkinje cell protein 4 [Source:HGNC Symbol;Acc:HGNC:8742] | 9.276320281 |
| WNT4 | Wnt family member 4 [Source:HGNC Symbol;Acc:HGNC:12783] | 8.532097107 |
| SHOC1 | shortage in chiasmata 1 [Source:HGNC Symbol;Acc:HGNC:26535] | 8.420806275 |
| TAFA4 | TAFA chemokine like family member 4 [Source:HGNC Symbol;Acc:HGNC:21591] | 8.149245337 |
| COL26A1 | collagen type XXVI alpha 1 chain [Source:HGNC Symbol;Acc:HGNC:18038] | 8.077315887 |
| KCNJ5 | potassium inwardly rectifying channel subfamily J member 5 [Source:HGNC Symbol;Acc:HGNC:6266] | 8.039007822 |
| RELN | reelin [Source:HGNC Symbol;Acc:HGNC:9957] | 7.991226893 |
| CDH2 | cadherin 2 [Source:HGNC Symbol;Acc:HGNC:1759] | 7.984328405 |
| NKD1 | NKD inhibitor of WNT signaling pathway 1 [Source:HGNC Symbol;Acc:HGNC:17045] | 7.895376799 |
| NTM | neurotrimin [Source:HGNC Symbol;Acc:HGNC:17941] | 7.678873775 |
| SOWAHB | sosondowah ankyrin repeat domain family member B [Source:HGNC Symbol;Acc:HGNC:32958] | 7.60993014 |
| ASTN1 | astrotactin 1 [Source:HGNC Symbol;Acc:HGNC:773] | 7.45411774 |
| RORB | RAR related orphan receptor B [Source:HGNC Symbol;Acc:HGNC:10259] | 7.359353752 |
| ATP2B3 | ATPase plasma membrane Ca2+ transporting 3 [Source:HGNC Symbol;Acc:HGNC:816] | 7.078249987 |
| TNFRSF19 | TNF receptor superfamily member 19 [Source:HGNC Symbol;Acc:HGNC:11915] | 7.016826873 |
| NEFM | neurofilament medium [Source:HGNC Symbol;Acc:HGNC:7734] | 6.810567501 |
| AARD | alanine and arginine rich domain containing protein [Source:HGNC Symbol;Acc:HGNC:33842] | 6.698896914 |
| TCF7 | transcription factor 7 [Source:HGNC Symbol;Acc:HGNC:11639] | 6.649692213 |
| LEF1 | lymphoid enhancer binding factor 1 [Source:HGNC Symbol;Acc:HGNC:6551] | 6.630420206 |
| CDO1 | cysteine dioxygenase type 1 [Source:HGNC Symbol;Acc:HGNC:1795] | 6.617185366 |
| ZCCHC12 | zinc finger CCHC-type containing 12 [Source:HGNC Symbol;Acc:HGNC:27273] | 6.59691251 |
| COLGALT2 | collagen beta(1-O)galactosyltransferase 2 [Source:HGNC Symbol;Acc:HGNC:16790] | 6.59538864 |
| PDE2A | phosphodiesterase 2A [Source:HGNC Symbol;Acc:HGNC:8777] | 6.538300715 |
| GABBR2 | gamma-aminobutyric acid type B receptor subunit 2 [Source:HGNC Symbol;Acc:HGNC:4507] | 6.506367306 |
| SPOCK1 | SPARC (osteonectin), cwcv and kazal like domains proteoglycan 1 [Source:HGNC Symbol;Acc:HGNC:11251] | 6.50347894 |
| ABCB4 | ATP binding cassette subfamily B member 4 [Source:HGNC Symbol;Acc:HGNC:45] | 6.358889849 |
| PDZRN3 | PDZ domain containing ring finger 3 [Source:HGNC Symbol;Acc:HGNC:17704] | 6.186865086 |
| KCNK2 | potassium two pore domain channel subfamily K member 2 [Source:HGNC Symbol;Acc:HGNC:6277] | 6.05458958 |
| NEFL | neurofilament light [Source:HGNC Symbol;Acc:HGNC:7739] | 5.895359702 |
| ZNF711 | zinc finger protein 711 [Source:HGNC Symbol;Acc:HGNC:13128] | 5.87694365 |
| CGNL1 | cingulin like 1 [Source:HGNC Symbol;Acc:HGNC:25931] | 5.848014451 |
| ADGRB1 | adhesion G protein-coupled receptor B1 [Source:HGNC Symbol;Acc:HGNC:943] | 5.844645113 |
| FGF12 | fibroblast growth factor 12 [Source:HGNC Symbol;Acc:HGNC:3668] | 5.809819869 |
| AFF3 | AF4/FMR2 family member 3 [Source:HGNC Symbol;Acc:HGNC:6473] | 5.768957296 |
| APCDD1 | APC down-regulated 1 [Source:HGNC Symbol;Acc:HGNC:15718] | 5.689692616 |
| GSTT2B | glutathione S-transferase theta 2B (gene/pseudogene) [Source:HGNC Symbol;Acc:HGNC:33437] | 5.671025201 |
| ASB4 | ankyrin repeat and SOCS box containing 4 [Source:HGNC Symbol;Acc:HGNC:16009] | 5.669144084 |
| RNF43 | ring finger protein 43 [Source:HGNC Symbol;Acc:HGNC:18505] | 5.612902675 |
| SV2A | synaptic vesicle glycoprotein 2A [Source:HGNC Symbol;Acc:HGNC:20566] | 5.585744766 |
| QPCT | glutaminyl-peptide cyclotransferase [Source:HGNC Symbol;Acc:HGNC:9753] | 5.548522975 |
| CABP7 | calcium binding protein 7 [Source:HGNC Symbol;Acc:HGNC:20834] | 5.541183823 |
| LPAR1 | lysophosphatidic acid receptor 1 [Source:HGNC Symbol;Acc:HGNC:3166] | 5.536118104 |
| BMP4 | bone morphogenetic protein 4 [Source:HGNC Symbol;Acc:HGNC:1071] | 5.459519786 |
| SLC44A5 | solute carrier family 44 member 5 [Source:HGNC Symbol;Acc:HGNC:28524] | 5.458577842 |
| VPREB3 | V-set pre-B cell surrogate light chain 3 [Source:HGNC Symbol;Acc:HGNC:12710] | 5.424329851 |
| LINC01537 | long intergenic non-protein coding RNA 1537 [Source:HGNC Symbol;Acc:HGNC:51301] | 5.414016888 |
| FRRS1L | ferric chelate reductase 1 like [Source:HGNC Symbol;Acc:HGNC:1362] | 5.381643283 |
| FAM169A | family with sequence similarity 169 member A [Source:HGNC Symbol;Acc:HGNC:29138] | 5.317024586 |
| RHBDL3 | rhomboid like 3 [Source:HGNC Symbol;Acc:HGNC:16502] | 5.310985156 |
| FAM181B | family with sequence similarity 181 member B [Source:HGNC Symbol;Acc:HGNC:28512] | 5.293014763 |
| MOXD1 | monooxygenase DBH like 1 [Source:HGNC Symbol;Acc:HGNC:21063] | 5.209185512 |
| HLF | HLF transcription factor, PAR bZIP family member [Source:HGNC Symbol;Acc:HGNC:4977] | 5.178293379 |
| TMEM200A | transmembrane protein 200A [Source:HGNC Symbol;Acc:HGNC:21075] | 5.101061906 |
| NSG1 | neuronal vesicle trafficking associated 1 [Source:HGNC Symbol;Acc:HGNC:18790] | 5.09706116 |
| BRINP2 | BMP/retinoic acid inducible neural specific 2 [Source:HGNC Symbol;Acc:HGNC:13746] | 5.072275714 |
| SLIT2 | slit guidance ligand 2 [Source:HGNC Symbol;Acc:HGNC:11086] | 5.056855805 |
| PLD5 | phospholipase D family member 5 [Source:HGNC Symbol;Acc:HGNC:26879] | 5.019127523 |
| SLC30A10 | solute carrier family 30 member 10 [Source:HGNC Symbol;Acc:HGNC:25355] | 5.018258917 |
| DACH1 | dachshund family transcription factor 1 [Source:HGNC Symbol;Acc:HGNC:2663] | 4.959632174 |
| PXYLP1 | 2-phosphoxylose phosphatase 1 [Source:HGNC Symbol;Acc:HGNC:26303] | 4.929205423 |
| CCDC170 | coiled-coil domain containing 170 [Source:HGNC Symbol;Acc:HGNC:21177] | 4.910118206 |
| NRXN2 | neurexin 2 [Source:HGNC Symbol;Acc:HGNC:8009] | 4.896016603 |
| MCOLN3 | mucolipin 3 [Source:HGNC Symbol;Acc:HGNC:13358] | 4.855148667 |
| DCN | decorin [Source:HGNC Symbol;Acc:HGNC:2705] | 4.845055415 |
| RASL10B | RAS like family 10 member B [Source:HGNC Symbol;Acc:HGNC:30295] | 4.829112611 |
| LSAMP | limbic system associated membrane protein [Source:HGNC Symbol;Acc:HGNC:6705] | 4.802146372 |
| TMEM200C | transmembrane protein 200C [Source:HGNC Symbol;Acc:HGNC:37208] | 4.79889476 |
| TNFSF4 | TNF superfamily member 4 [Source:HGNC Symbol;Acc:HGNC:11934] | 4.742475031 |
| LINC02593 | long intergenic non-protein coding RNA 2593 [Source:HGNC Symbol;Acc:HGNC:53933] | 4.683832798 |
| P3H3 | prolyl 3-hydroxylase 3 [Source:HGNC Symbol;Acc:HGNC:19318] | 4.675995953 |
| AXIN2 | axin 2 [Source:HGNC Symbol;Acc:HGNC:904] | 4.632330498 |
| TUBBP5 | tubulin beta pseudogene 5 [Source:HGNC Symbol;Acc:HGNC:23674] | 4.60989891 |
| VCAN | versican [Source:HGNC Symbol;Acc:HGNC:2464] | 4.575078298 |
| HOXB9 | homeobox B9 [Source:HGNC Symbol;Acc:HGNC:5120] | 4.567042128 |
| SYTL2 | synaptotagmin like 2 [Source:HGNC Symbol;Acc:HGNC:15585] | 4.564036553 |
| AF106564.1 | novel transcript, antisense to NEFM | 4.554082311 |
| ETV4 | ETS variant transcription factor 4 [Source:HGNC Symbol;Acc:HGNC:3493] | 4.551940006 |
| SHROOM3 | shroom family member 3 [Source:HGNC Symbol;Acc:HGNC:30422] | 4.550764173 |
| UGCG | UDP-glucose ceramide glucosyltransferase [Source:HGNC Symbol;Acc:HGNC:12524] | 4.542428054 |

| | | |
|---|---|---|
| ABCD2 | ATP binding cassette subfamily D member 2 [Source:HGNC Symbol;Acc:HGNC:66] | 4.536893537 |
| TSPAN8 | tetraspanin 8 [Source:HGNC Symbol;Acc:HGNC:11855] | 4.487478615 |
| AC010501.2 | novel transcript, antisense to FAM169A | 4.471136683 |
| ESRRG | estrogen related receptor gamma [Source:HGNC Symbol;Acc:HGNC:3474] | 4.469525251 |
| IGSF5 | immunoglobulin superfamily member 5 [Source:HGNC Symbol;Acc:HGNC:5952] | 4.462607159 |
| UBE2QL1 | ubiquitin conjugating enzyme E2 Q family like 1 [Source:HGNC Symbol;Acc:HGNC:37269] | 4.449074519 |
| MYH7B | myosin heavy chain 7B [Source:HGNC Symbol;Acc:HGNC:15906] | 4.44096187 |
| ANO4 | anoctamin 4 [Source:HGNC Symbol;Acc:HGNC:23837] | 4.428175052 |
| DPP10 | dipeptidyl peptidase like 10 [Source:HGNC Symbol;Acc:HGNC:20823] | 4.427363781 |
| SLC2A4 | solute carrier family 2 member 4 [Source:HGNC Symbol;Acc:HGNC:11009] | 4.419777962 |
| AGTR1 | angiotensin II receptor type 1 [Source:HGNC Symbol;Acc:HGNC:336] | 4.393545171 |
| HSD3B2 | hydroxy-delta-5-steroid dehydrogenase, 3 beta- and steroid delta-isomerase 2 [Source:HGNC Symbol;Acc:HGNC:5218] | 4.392446998 |
| FAM135B | family with sequence similarity 135 member B [Source:HGNC Symbol;Acc:HGNC:28029] | 4.383886367 |
| CACNA2D1 | calcium voltage-gated channel auxiliary subunit alpha2delta 1 [Source:HGNC Symbol;Acc:HGNC:1399] | 4.334354048 |
| ATP10A | ATPase phospholipid transporting 10A (putative) [Source:HGNC Symbol;Acc:HGNC:13542] | 4.319443586 |
| PGM5 | phosphoglucomutase 5 [Source:HGNC Symbol;Acc:HGNC:8908] | 4.307692919 |
| ECEL1 | endothelin converting enzyme like 1 [Source:HGNC Symbol;Acc:HGNC:3147] | 4.297255605 |
| PEG10 | paternally expressed 10 [Source:HGNC Symbol;Acc:HGNC:14005] | 4.296466029 |
| PTPN7 | protein tyrosine phosphatase non-receptor type 7 [Source:HGNC Symbol;Acc:HGNC:9659] | 4.295422386 |
| MERTK | MER proto-oncogene, tyrosine kinase [Source:HGNC Symbol;Acc:HGNC:7027] | 4.262026588 |
| GOLGA7B | golgin A7 family member B [Source:HGNC Symbol;Acc:HGNC:31668] | 4.252937006 |
| MCF2 | MCF.2 cell line derived transforming sequence [Source:HGNC Symbol;Acc:HGNC:6940] | 4.230788237 |
| SP5 | Sp5 transcription factor [Source:HGNC Symbol;Acc:HGNC:14529] | 4.221925611 |
| CERS1 | ceramide synthase 1 [Source:HGNC Symbol;Acc:HGNC:14253] | 4.212903723 |
| ASGR1 | asialoglycoprotein receptor 1 [Source:HGNC Symbol;Acc:HGNC:742] | 4.211422587 |
| VGF | VGF nerve growth factor inducible [Source:HGNC Symbol;Acc:HGNC:12684] | 4.1992842 |
| LRCH2 | leucine rich repeats and calponin homology domain containing 2 [Source:HGNC Symbol;Acc:HGNC:29292] | 4.176965999 |
| CADM1 | cell adhesion molecule 1 [Source:HGNC Symbol;Acc:HGNC:5951] | 4.156518994 |
| ST6GALNAC5 | ST6 N-acetylgalactosaminide alpha-2,6-sialyltransferase 5 [Source:HGNC Symbol;Acc:HGNC:19342] | 4.151804052 |
| RIMS2 | regulating synaptic membrane exocytosis 2 [Source:HGNC Symbol;Acc:HGNC:17283] | 4.116882656 |
| DKK3 | dickkopf WNT signaling pathway inhibitor 3 [Source:HGNC Symbol;Acc:HGNC:2893] | 4.103093305 |
| FGF20 | fibroblast growth factor 20 [Source:HGNC Symbol;Acc:HGNC:3677] | 4.101910938 |
| FST | follistatin [Source:HGNC Symbol;Acc:HGNC:3971] | 4.090806076 |
| CHRD | chordin [Source:HGNC Symbol;Acc:HGNC:1949] | 4.072099984 |
| ABR | ABR activator of RhoGEF and GTPase [Source:HGNC Symbol;Acc:HGNC:81] | 4.056074785 |
| ACOX2 | acyl-CoA oxidase 2 [Source:HGNC Symbol;Acc:HGNC:120] | 4.049839956 |
| CPB1 | carboxypeptidase B1 [Source:HGNC Symbol;Acc:HGNC:2299] | 4.037100922 |
| PLXNB3 | plexin B3 [Source:HGNC Symbol;Acc:HGNC:9105] | 4.023695457 |
| TGFB2 | transforming growth factor beta 2 [Source:HGNC Symbol;Acc:HGNC:11768] | 3.980640371 |
| SLC35F3 | solute carrier family 35 member F3 [Source:HGNC Symbol;Acc:HGNC:23616] | 3.958342152 |
| GPR162 | G protein-coupled receptor 162 [Source:HGNC Symbol;Acc:HGNC:16693] | 3.953060734 |
| PLCL2 | phospholipase C like 2 [Source:HGNC Symbol;Acc:HGNC:9064] | 3.938734613 |
| KIF7 | kinesin family member 7 [Source:HGNC Symbol;Acc:HGNC:30497] | 3.911199003 |
| WNK2 | WNK lysine deficient protein kinase 2 [Source:HGNC Symbol;Acc:HGNC:14542] | 3.893686613 |
| GFAP | glial fibrillary acidic protein [Source:HGNC Symbol;Acc:HGNC:4235] | 3.87159479 |
| SAMD11 | sterile alpha motif domain containing 11 [Source:HGNC Symbol;Acc:HGNC:28706] | 3.849088641 |
| BANK1 | B cell scaffold protein with ankyrin repeats 1 [Source:HGNC Symbol;Acc:HGNC:18233] | 3.846041724 |
| PNMA3 | PNMA family member 3 [Source:HGNC Symbol;Acc:HGNC:18742] | 3.831059626 |
| RPRM | reprimo, TP53 dependent G2 arrest mediator homolog [Source:HGNC Symbol;Acc:HGNC:24201] | 3.789072273 |
| TPBG | trophoblast glycoprotein [Source:HGNC Symbol;Acc:HGNC:12004] | 3.778716539 |
| RAB38 | RAB38, member RAS oncogene family [Source:HGNC Symbol;Acc:HGNC:9776] | 3.770348774 |
| LINC01116 | long intergenic non-protein coding RNA 1116 [Source:HGNC Symbol;Acc:HGNC:49259] | 3.763372135 |
| UNC5C | unc-5 netrin receptor C [Source:HGNC Symbol;Acc:HGNC:12569] | 3.750190385 |
| LGI3 | leucine rich repeat LGI family member 3 [Source:HGNC Symbol;Acc:HGNC:18711] | 3.74534925 |
| ADAMTSL1 | ADAMTS like 1 [Source:HGNC Symbol;Acc:HGNC:14632] | 3.740056394 |
| AC078880.3 | novel transcript | 3.735796463 |
| FUT1 | fucosyltransferase 1 (H blood group) [Source:HGNC Symbol;Acc:HGNC:4012] | 3.729352252 |
| SLC12A8 | solute carrier family 12 member 8 [Source:HGNC Symbol;Acc:HGNC:15595] | 3.720634686 |
| SLC22A3 | solute carrier family 22 member 3 [Source:HGNC Symbol;Acc:HGNC:10967] | 3.714524786 |
| TMEM132E | transmembrane protein 132E [Source:HGNC Symbol;Acc:HGNC:26991] | 3.712154848 |
| STEAP2 | STEAP2 metalloreductase [Source:HGNC Symbol;Acc:HGNC:17885] | 3.65402769 |
| CILP2 | cartilage intermediate layer protein 2 [Source:HGNC Symbol;Acc:HGNC:24213] | 3.630187221 |
| KRT222 | keratin 222 [Source:HGNC Symbol;Acc:HGNC:28695] | 3.629239963 |
| DAB2 | DAB adaptor protein 2 [Source:HGNC Symbol;Acc:HGNC:2662] | 3.594604088 |
| ALDH1L1 | aldehyde dehydrogenase 1 family member L1 [Source:HGNC Symbol;Acc:HGNC:3978] | 3.592053801 |
| MIR100HG | mir-100-let-7a-2-mir-125b-1 cluster host gene [Source:HGNC Symbol;Acc:HGNC:39522] | 3.584244266 |
| NFATC4 | nuclear factor of activated T cells 4 [Source:HGNC Symbol;Acc:HGNC:7778] | 3.57075142 |
| HMGA2 | high mobility group AT-hook 2 [Source:HGNC Symbol;Acc:HGNC:5009] | 3.563448456 |
| BICC1 | BicC family RNA binding protein 1 [Source:HGNC Symbol;Acc:HGNC:19351] | 3.548432437 |
| LAMC3 | laminin subunit gamma 3 [Source:HGNC Symbol;Acc:HGNC:6494] | 3.547045299 |
| SCARA3 | scavenger receptor class A member 3 [Source:HGNC Symbol;Acc:HGNC:19000] | 3.527053799 |
| ACSL6 | acyl-CoA synthetase long chain family member 6 [Source:HGNC Symbol;Acc:HGNC:16496] | 3.517165875 |
| ARHGEF4 | Rho guanine nucleotide exchange factor 4 [Source:HGNC Symbol;Acc:HGNC:684] | 3.515762991 |
| KCNN2 | potassium calcium-activated channel subfamily N member 2 [Source:HGNC Symbol;Acc:HGNC:6291] | 3.513912991 |
| ITGA2 | integrin subunit alpha 2 [Source:HGNC Symbol;Acc:HGNC:6137] | 3.505574929 |
| UBXN10 | UBX domain protein 10 [Source:HGNC Symbol;Acc:HGNC:26354] | 3.494935718 |
| AC103702.2 | novel transcript | 3.491879705 |
| MDK | midkine [Source:HGNC Symbol;Acc:HGNC:6972] | 3.482139837 |
| IFITM10 | interferon induced transmembrane protein 10 [Source:HGNC Symbol;Acc:HGNC:40022] | 3.472838116 |
| ADAM22 | ADAM metallopeptidase domain 22 [Source:HGNC Symbol;Acc:HGNC:201] | 3.470823364 |
| MC2R | melanocortin 2 receptor [Source:HGNC Symbol;Acc:HGNC:6930] | 3.447979403 |
| FAM171B | family with sequence similarity 171 member B [Source:HGNC Symbol;Acc:HGNC:29412] | 3.432070929 |
| ALDH1A2 | aldehyde dehydrogenase 1 family member A2 [Source:HGNC Symbol;Acc:HGNC:15472] | 3.429471745 |
| NAGS | N-acetylglutamate synthase [Source:HGNC Symbol;Acc:HGNC:17996] | 3.407611634 |
| RUNX1T1 | RUNX1 partner transcriptional co-repressor 1 [Source:HGNC Symbol;Acc:HGNC:1535] | 3.402333968 |
| MAP7D2 | MAP7 domain containing 2 [Source:HGNC Symbol;Acc:HGNC:25899] | 3.398586024 |
| CA11 | carbonic anhydrase 11 [Source:HGNC Symbol;Acc:HGNC:1370] | 3.396472239 |

| | | |
|---|---|---|
| AL136962.1 | long intergenic non-protein coding RNA 540 | 3.396089218 |
| BDNF | brain derived neurotrophic factor [Source:HGNC Symbol;Acc:HGNC:1033] | 3.384810998 |
| SLC4A8 | solute carrier family 4 member 8 [Source:HGNC Symbol;Acc:HGNC:11034] | 3.353501372 |
| NR4A3 | nuclear receptor subfamily 4 group A member 3 [Source:HGNC Symbol;Acc:HGNC:7982] | 3.33717232 |
| RASAL1 | RAS protein activator like 1 [Source:HGNC Symbol;Acc:HGNC:9873] | 3.320189605 |
| KHDRBS3 | KH RNA binding domain containing, signal transduction associated 3 [Source:HGNC Symbol;Acc:HGNC:18117] | 3.317878872 |
| SV2B | synaptic vesicle glycoprotein 2B [Source:HGNC Symbol;Acc:HGNC:16874] | 3.317564987 |
| CCDC73 | coiled-coil domain containing 73 [Source:HGNC Symbol;Acc:HGNC:23261] | 3.317311829 |
| LRATD1 | LRAT domain containing 1 [Source:HGNC Symbol;Acc:HGNC:20743] | 3.315679757 |
| TMEM221 | transmembrane protein 221 [Source:HGNC Symbol;Acc:HGNC:21943] | 3.30986353 |
| TMEM108 | transmembrane protein 108 [Source:HGNC Symbol;Acc:HGNC:28451] | 3.300685496 |
| CIB4 | calcium and integrin binding family member 4 [Source:HGNC Symbol;Acc:HGNC:33703] | 3.299349753 |
| SPON1 | spondin 1 [Source:HGNC Symbol;Acc:HGNC:11252] | 3.294723614 |
| LARP6 | La ribonucleoprotein 6, translational regulator [Source:HGNC Symbol;Acc:HGNC:24012] | 3.288374352 |
| GATA3 | GATA binding protein 3 [Source:HGNC Symbol;Acc:HGNC:4172] | 3.287684882 |
| PRRX1 | paired related homeobox 1 [Source:HGNC Symbol;Acc:HGNC:9142] | 3.285112702 |
| NPTX1 | neuronal pentraxin 1 [Source:HGNC Symbol;Acc:HGNC:7952] | 3.28505917 |
| LRRN2 | leucine rich repeat neuronal 2 [Source:HGNC Symbol;Acc:HGNC:16914] | 3.284457696 |
| SRPX | sushi repeat containing protein X-linked [Source:HGNC Symbol;Acc:HGNC:11309] | 3.277388925 |
| CSRP2 | cysteine and glycine rich protein 2 [Source:HGNC Symbol;Acc:HGNC:2470] | 3.273936181 |
| TMEM25 | transmembrane protein 25 [Source:HGNC Symbol;Acc:HGNC:25890] | 3.26230189 |
| WNT3 | Wnt family member 3 [Source:HGNC Symbol;Acc:HGNC:12782] | 3.246275036 |
| SLC22A17 | solute carrier family 22 member 17 [Source:HGNC Symbol;Acc:HGNC:23095] | 3.238583129 |
| INPP4B | inositol polyphosphate-4-phosphatase type II B [Source:HGNC Symbol;Acc:HGNC:6075] | 3.232259969 |
| CTHRC1 | collagen triple helix repeat containing 1 [Source:HGNC Symbol;Acc:HGNC:18831] | 3.22589785 |
| PHLDA1 | pleckstrin homology like domain family A member 1 [Source:HGNC Symbol;Acc:HGNC:8933] | 3.221197075 |
| CCDC184 | coiled-coil domain containing 184 [Source:HGNC Symbol;Acc:HGNC:33749] | 3.219094254 |
| PRKD3 | protein kinase D3 [Source:HGNC Symbol;Acc:HGNC:9408] | 3.206893556 |
| C11orf45 | chromosome 11 open reading frame 45 [Source:HGNC Symbol;Acc:HGNC:28584] | 3.206810226 |
| ETV5 | ETS variant transcription factor 5 [Source:HGNC Symbol;Acc:HGNC:3494] | 3.195045095 |
| RTN4R | reticulon 4 receptor [Source:HGNC Symbol;Acc:HGNC:18601] | 3.192028782 |
| AATK | apoptosis associated tyrosine kinase [Source:HGNC Symbol;Acc:HGNC:21] | 3.186210375 |
| PITX1 | paired like homeodomain 1 [Source:HGNC Symbol;Acc:HGNC:9004] | 3.171611441 |
| HYAL1 | hyaluronidase 1 [Source:HGNC Symbol;Acc:HGNC:5320] | 3.16421807 |
| SLC16A2 | solute carrier family 16 member 2 [Source:HGNC Symbol;Acc:HGNC:10923] | 3.160515269 |
| SLC6A9 | solute carrier family 6 member 9 [Source:HGNC Symbol;Acc:HGNC:11056] | 3.158840236 |
| AMOT | angiomotin [Source:HGNC Symbol;Acc:HGNC:17810] | 3.134783393 |
| PLPP2 | phospholipid phosphatase 2 [Source:HGNC Symbol;Acc:HGNC:9230] | 3.133352954 |
| CD5 | CD5 molecule [Source:HGNC Symbol;Acc:HGNC:1685] | 3.129506173 |
| EML6 | EMAP like 6 [Source:HGNC Symbol;Acc:HGNC:35412] | 3.120340354 |
| PRSS35 | serine protease 35 [Source:HGNC Symbol;Acc:HGNC:21387] | 3.119982662 |
| JAM2 | junctional adhesion molecule 2 [Source:HGNC Symbol;Acc:HGNC:14686] | 3.119235943 |
| HS3ST3B1 | heparan sulfate-glucosamine 3-sulfotransferase 3B1 [Source:HGNC Symbol;Acc:HGNC:5198] | 3.118166615 |
| COL4A3 | collagen type IV alpha 3 chain [Source:HGNC Symbol;Acc:HGNC:2204] | 3.106153221 |
| ATP2A3 | ATPase sarcoplasmic/endoplasmic reticulum Ca2+ transporting 3 [Source:HGNC Symbol;Acc:HGNC:813] | 3.102200845 |
| CACNB4 | calcium voltage-gated channel auxiliary subunit beta 4 [Source:HGNC Symbol;Acc:HGNC:1404] | 3.10134349 |
| TNFSF13B | TNF superfamily member 13b [Source:HGNC Symbol;Acc:HGNC:11929] | 3.083959251 |
| NANOGP1 | Nanog homeobox pseudogene 1 [Source:HGNC Symbol;Acc:HGNC:23099] | 3.083422748 |
| NFASC | neurofascin [Source:HGNC Symbol;Acc:HGNC:29866] | 3.082066784 |
| SLC47A1 | solute carrier family 47 member 1 [Source:HGNC Symbol;Acc:HGNC:25588] | 3.073437312 |
| ELOVL4 | ELOVL fatty acid elongase 4 [Source:HGNC Symbol;Acc:HGNC:14415] | 3.07185023 |
| VSTM4 | V-set and transmembrane domain containing 4 [Source:HGNC Symbol;Acc:HGNC:26470] | 3.071838635 |
| SMO | smoothened, frizzled class receptor [Source:HGNC Symbol;Acc:HGNC:11119] | 3.065417985 |
| ALCAM | activated leukocyte cell adhesion molecule [Source:HGNC Symbol;Acc:HGNC:400] | 3.058330556 |
| SEMA3C | semaphorin 3C [Source:HGNC Symbol;Acc:HGNC:10725] | 3.057838153 |
| UNC79 | unc-79 homolog, NALCN channel complex subunit [Source:HGNC Symbol;Acc:HGNC:19966] | 3.054916859 |
| CRABP2 | cellular retinoic acid binding protein 2 [Source:HGNC Symbol;Acc:HGNC:2339] | 3.034048234 |
| JPH2 | junctophilin 2 [Source:HGNC Symbol;Acc:HGNC:14202] | 3.028099555 |
| SERINC5 | serine incorporator 5 [Source:HGNC Symbol;Acc:HGNC:18825] | 3.026909224 |
| SORBS2 | sorbin and SH3 domain containing 2 [Source:HGNC Symbol;Acc:HGNC:24098] | 3.022167142 |
| RARG | retinoic acid receptor gamma [Source:HGNC Symbol;Acc:HGNC:9866] | 3.021829035 |
| PLEKHF1 | pleckstrin homology and FYVE domain containing 1 [Source:HGNC Symbol;Acc:HGNC:20764] | 3.010582397 |
| ATP1B2 | ATPase Na+/K+ transporting subunit beta 2 [Source:HGNC Symbol;Acc:HGNC:805] | 3.0084944 |
| CCND1 | cyclin D1 [Source:HGNC Symbol;Acc:HGNC:1582] | 3.008442635 |
| CLGN | calmegin [Source:HGNC Symbol;Acc:HGNC:2060] | 3.006082526 |
| FAM149A | family with sequence similarity 149 member A [Source:HGNC Symbol;Acc:HGNC:24527] | 2.99852404 |
| RGS14 | regulator of G protein signaling 14 [Source:HGNC Symbol;Acc:HGNC:9996] | 2.991397068 |
| DAAM2 | dishevelled associated activator of morphogenesis 2 [Source:HGNC Symbol;Acc:HGNC:18143] | 2.971777151 |
| GNAL | G protein subunit alpha L [Source:HGNC Symbol;Acc:HGNC:4388] | 2.970271031 |
| LINC01616 | long intergenic non-protein coding RNA 1616 [Source:HGNC Symbol;Acc:HGNC:51900] | 2.970084543 |
| HEPH | hephaestin [Source:HGNC Symbol;Acc:HGNC:4866] | 2.969040974 |
| PDE5A | phosphodiesterase 5A [Source:HGNC Symbol;Acc:HGNC:8784] | 2.967107414 |
| SYTL5 | synaptotagmin like 5 [Source:HGNC Symbol;Acc:HGNC:15589] | 2.96007372 |
| FAM110B | family with sequence similarity 110 member B [Source:HGNC Symbol;Acc:HGNC:28587] | 2.957861462 |
| SBSPON | somatomedin B and thrombospondin type 1 domain containing [Source:HGNC Symbol;Acc:HGNC:30362] | 2.95093422 |
| CAPN5 | calpain 5 [Source:HGNC Symbol;Acc:HGNC:1482] | 2.94979603 |
| INSYN1 | inhibitory synaptic factor 1 [Source:HGNC Symbol;Acc:HGNC:33753] | 2.942737258 |
| HR | HR lysine demethylase and nuclear receptor corepressor [Source:HGNC Symbol;Acc:HGNC:5172] | 2.937932427 |
| CACNB2 | calcium voltage-gated channel auxiliary subunit beta 2 [Source:HGNC Symbol;Acc:HGNC:1402] | 2.932973065 |
| KREMEN2 | kringle containing transmembrane protein 2 [Source:HGNC Symbol;Acc:HGNC:18797] | 2.931015877 |
| AL049838.1 | novel transcript, overlapping C14orf37 | 2.930122604 |
| RAET1G | retinoic acid early transcript 1G [Source:HGNC Symbol;Acc:HGNC:16795] | 2.926386489 |
| EFNA5 | ephrin A5 [Source:HGNC Symbol;Acc:HGNC:3225] | 2.925287463 |
| RBPMS2 | RNA binding protein, mRNA processing factor 2 [Source:HGNC Symbol;Acc:HGNC:19098] | 2.922238417 |
| PPM1E | protein phosphatase, Mg2+/Mn2+ dependent 1E [Source:HGNC Symbol;Acc:HGNC:19322] | 2.916762301 |
| PNMA6A | PNMA family member 6A [Source:HGNC Symbol;Acc:HGNC:28248] | 2.912594662 |
| RNF217 | ring finger protein 217 [Source:HGNC Symbol;Acc:HGNC:21487] | 2.912358413 |

| | | |
|---|---|---|
| ADGRD1 | adhesion G protein-coupled receptor D1 [Source:HGNC Symbol;Acc:HGNC:19893] | 2.91111432 |
| FAM110C | family with sequence similarity 110 member C [Source:HGNC Symbol;Acc:HGNC:33340] | 2.910322624 |
| NPNT | nephronectin [Source:HGNC Symbol;Acc:HGNC:27405] | 2.908321711 |
| ERN1 | endoplasmic reticulum to nucleus signaling 1 [Source:HGNC Symbol;Acc:HGNC:3449] | 2.894596079 |
| ISLR2 | immunoglobulin superfamily containing leucine rich repeat 2 [Source:HGNC Symbol;Acc:HGNC:29286] | 2.88638995 |
| GPR61 | G protein-coupled receptor 61 [Source:HGNC Symbol;Acc:HGNC:13300] | 2.881038169 |
| PYGO1 | pygopus family PHD finger 1 [Source:HGNC Symbol;Acc:HGNC:30256] | 2.861441215 |
| MAPK8IP2 | mitogen-activated protein kinase 8 interacting protein 2 [Source:HGNC Symbol;Acc:HGNC:6883] | 2.855893038 |
| CLDN1 | claudin 1 [Source:HGNC Symbol;Acc:HGNC:2032] | 2.850060981 |
| DTX1 | deltex E3 ubiquitin ligase 1 [Source:HGNC Symbol;Acc:HGNC:3060] | 2.845273518 |
| MINAR1 | membrane integral NOTCH2 associated receptor 1 [Source:HGNC Symbol;Acc:HGNC:29172] | 2.845027326 |
| NUDT11 | nudix hydrolase 11 [Source:HGNC Symbol;Acc:HGNC:18011] | 2.844089949 |
| RAB39B | RAB39B, member RAS oncogene family [Source:HGNC Symbol;Acc:HGNC:16499] | 2.843534296 |
| MFAP4 | microfibril associated protein 4 [Source:HGNC Symbol;Acc:HGNC:7035] | 2.840046095 |
| KIF5A | kinesin family member 5A [Source:HGNC Symbol;Acc:HGNC:6323] | 2.83465663 |
| LY75 | lymphocyte antigen 75 [Source:HGNC Symbol;Acc:HGNC:6729] | 2.834630388 |
| ADH1B | alcohol dehydrogenase 1B (class I), beta polypeptide [Source:HGNC Symbol;Acc:HGNC:250] | 2.834502458 |
| GPRC5C | G protein-coupled receptor class C group 5 member C [Source:HGNC Symbol;Acc:HGNC:13309] | 2.825424834 |
| ACAN | aggrecan [Source:HGNC Symbol;Acc:HGNC:319] | 2.823532019 |
| EVC | EvC ciliary complex subunit 1 [Source:HGNC Symbol;Acc:HGNC:3497] | 2.82351937 |
| KAZN | kazrin, periplakin interacting protein [Source:HGNC Symbol;Acc:HGNC:29173] | 2.815047916 |
| FBXL7 | F-box and leucine rich repeat protein 7 [Source:HGNC Symbol;Acc:HGNC:13604] | 2.814332582 |
| GRB14 | growth factor receptor bound protein 14 [Source:HGNC Symbol;Acc:HGNC:4565] | 2.813400405 |
| PRR7 | proline rich 7, synaptic [Source:HGNC Symbol;Acc:HGNC:28130] | 2.805984642 |
| ATL1 | atlastin GTPase 1 [Source:HGNC Symbol;Acc:HGNC:11231] | 2.794223373 |
| CYP11B2 | cytochrome P450 family 11 subfamily B member 2 [Source:HGNC Symbol;Acc:HGNC:2592] | 2.79125585 |
| ADAMTS9-AS2 | ADAMTS9 antisense RNA 2 [Source:HGNC Symbol;Acc:HGNC:42435] | 2.789765117 |
| AC079949.2 | novel transcript | 2.787696091 |
| KCNG1 | potassium voltage-gated channel modifier subfamily G member 1 [Source:HGNC Symbol;Acc:HGNC:6248] | 2.786856296 |
| DNM3 | dynamin 3 [Source:HGNC Symbol;Acc:HGNC:29125] | 2.785794748 |
| SDK2 | sidekick cell adhesion molecule 2 [Source:HGNC Symbol;Acc:HGNC:19308] | 2.784231286 |
| PARVB | parvin beta [Source:HGNC Symbol;Acc:HGNC:14653] | 2.783173456 |
| ARG2 | arginase 2 [Source:HGNC Symbol;Acc:HGNC:664] | 2.767962118 |
| RHBG | Rh family B glycoprotein (gene/pseudogene) [Source:HGNC Symbol;Acc:HGNC:14572] | 2.763854586 |
| ATRNL1 | attractin like 1 [Source:HGNC Symbol;Acc:HGNC:29063] | 2.75506664 |
| PTGIS | prostaglandin I2 synthase [Source:HGNC Symbol;Acc:HGNC:9603] | 2.754263671 |
| OPRL1 | opioid related nociceptin receptor 1 [Source:HGNC Symbol;Acc:HGNC:8155] | 2.747288338 |
| LUM | lumican [Source:HGNC Symbol;Acc:HGNC:6724] | 2.744567441 |
| HOXB8 | homeobox B8 [Source:HGNC Symbol;Acc:HGNC:5119] | 2.741731147 |
| SPTLC3 | serine palmitoyltransferase long chain base subunit 3 [Source:HGNC Symbol;Acc:HGNC:16253] | 2.740615962 |
| LAMA2 | laminin subunit alpha 2 [Source:HGNC Symbol;Acc:HGNC:6482] | 2.740560949 |
| ASIC1 | acid sensing ion channel subunit 1 [Source:HGNC Symbol;Acc:HGNC:100] | 2.736871448 |
| SRCIN1 | SRC kinase signaling inhibitor 1 [Source:HGNC Symbol;Acc:HGNC:29506] | 2.736467056 |
| EFNA3 | ephrin A3 [Source:HGNC Symbol;Acc:HGNC:3223] | 2.736027496 |
| HSH2D | hematopoietic SH2 domain containing [Source:HGNC Symbol;Acc:HGNC:24920] | 2.734715163 |
| ID2 | inhibitor of DNA binding 2 [Source:HGNC Symbol;Acc:HGNC:5361] | 2.731855701 |
| AC005052.1 | TEC | 2.722565114 |
| SLC44A4 | solute carrier family 44 member 4 [Source:HGNC Symbol;Acc:HGNC:13941] | 2.715167874 |
| ETV1 | ETS variant transcription factor 1 [Source:HGNC Symbol;Acc:HGNC:3490] | 2.710317151 |
| PDK4 | pyruvate dehydrogenase kinase 4 [Source:HGNC Symbol;Acc:HGNC:8812] | 2.708258219 |
| TWSG1 | twisted gastrulation BMP signaling modulator 1 [Source:HGNC Symbol;Acc:HGNC:12429] | 2.699430046 |
| CDHR1 | cadherin related family member 1 [Source:HGNC Symbol;Acc:HGNC:14550] | 2.699269044 |
| LRRC17 | leucine rich repeat containing 17 [Source:HGNC Symbol;Acc:HGNC:16895] | 2.689449039 |
| IZUMO1 | izumo sperm-egg fusion 1 [Source:HGNC Symbol;Acc:HGNC:28539] | 2.676580183 |
| GPC4 | glypican 4 [Source:HGNC Symbol;Acc:HGNC:4452] | 2.673322808 |
| WDFY3-AS2 | WDFY3 antisense RNA 2 [Source:HGNC Symbol;Acc:HGNC:21603] | 2.650891431 |
| B3GNT9 | UDP-GlcNAc:betaGal beta-1,3-N-acetylglucosaminyltransferase 9 [Source:HGNC Symbol;Acc:HGNC:28714] | 2.642132894 |
| LSP1 | lymphocyte specific protein 1 [Source:HGNC Symbol;Acc:HGNC:6707] | 2.64000361 |
| KLHDC8A | kelch domain containing 8A [Source:HGNC Symbol;Acc:HGNC:25573] | 2.634699537 |
| IGHG1 | immunoglobulin heavy constant gamma 1 (G1m marker) [Source:HGNC Symbol;Acc:HGNC:5525] | 2.63395816 |
| RNF128 | ring finger protein 128 [Source:HGNC Symbol;Acc:HGNC:21153] | 2.608304983 |
| RPSAP70 | ribosomal protein SA pseudogene 70 [Source:HGNC Symbol;Acc:HGNC:51923] | 2.602362289 |
| AL139220.2 | novel transcript | 2.596280539 |
| SEMA4G | semaphorin 4G [Source:HGNC Symbol;Acc:HGNC:10735] | 2.585604789 |
| RASGEF1A | RasGEF domain family member 1A [Source:HGNC Symbol;Acc:HGNC:24246] | 2.581225911 |
| FLRT2 | fibronectin leucine rich transmembrane protein 2 [Source:HGNC Symbol;Acc:HGNC:3761] | 2.563045696 |
| SESN3 | sestrin 3 [Source:HGNC Symbol;Acc:HGNC:23060] | 2.558442689 |
| KCNMB4 | potassium calcium-activated channel subfamily M regulatory beta subunit 4 [Source:HGNC Symbol;Acc:HGNC:6289] | 2.539602592 |
| ERN2 | endoplasmic reticulum to nucleus signaling 2 [Source:HGNC Symbol;Acc:HGNC:16942] | 2.537724229 |
| TEC | tec protein tyrosine kinase [Source:HGNC Symbol;Acc:HGNC:11719] | 2.535657547 |
| WDR63 | WD repeat domain 63 [Source:HGNC Symbol;Acc:HGNC:30711] | 2.530274604 |
| MAP6 | microtubule associated protein 6 [Source:HGNC Symbol;Acc:HGNC:6868] | 2.528510817 |
| PRICKLE1 | prickle planar cell polarity protein 1 [Source:HGNC Symbol;Acc:HGNC:17019] | 2.528169594 |
| THNSL2 | threonine synthase like 2 [Source:HGNC Symbol;Acc:HGNC:25602] | 2.525314287 |
| IGKC | immunoglobulin kappa constant [Source:HGNC Symbol;Acc:HGNC:5716] | 2.523864896 |
| PCDHGB4 | protocadherin gamma subfamily B, 4 [Source:HGNC Symbol;Acc:HGNC:8711] | 2.514632776 |
| LIN7A | lin-7 homolog A, crumbs cell polarity complex component [Source:HGNC Symbol;Acc:HGNC:17787] | 2.513009823 |
| CDK6 | cyclin dependent kinase 6 [Source:HGNC Symbol;Acc:HGNC:1777] | 2.506890234 |
| MYO5B | myosin VB [Source:HGNC Symbol;Acc:HGNC:7603] | 2.50618462 |
| HS3ST1 | heparan sulfate-glucosamine 3-sulfotransferase 1 [Source:HGNC Symbol;Acc:HGNC:5194] | 2.503288908 |
| SSPO | SCO-spondin [Source:HGNC Symbol;Acc:HGNC:21998] | 2.500558646 |
