# Supplementary material for: Targeting Oncogenic Wnt/β-Catenin Signaling in Adrenocortical Carcinoma Disrupts ECM Expression and Impairs Tumor Growth
Source: Cancers (Basel). 2023 Jul 10;15(14):3559. doi: 10.3390/cancers15143559 (PMC10377252; doi:10.3390/cancers15143559)
Supplement: Supplementary file 1 [file cancers-15-03559-s001.zip › Supplementary Table S2 - Oligonucleotides used in this study.pdf]

Supplementary Table S2 - Oligonucleotides used in this study

Gene	Forward Primer 5' -> 3'	Reverse Primer 5' -> 3'
HPRT1	TGACACTGGCAAAACAATGCA	GGTCCTTTTCACCAGCAAGCT
AXIN2	AAGTGCAAACTTTCGCCAAC	ACAGGATCGCTCCTCTTGAA
LEF1	CTTTATCCAGGCTGGTCTGC	TCGTTTTCCACCATGTTTCA
APCDD1	ATGCCACCCAGAGGATGTTC	GATGGTCAGGTCTGCCTTTG
COL11A1	GACTATCCCCTCTTCAGAACTG	CTTCTATCAAGTGGTTTCGTGGTTT
COL26A1	CAGCAGCTGAGAGAGGCCCT	GCCACCCCTCTTCATCTTGAG
ITAG2	AACTCTTTGGATTTGCGTGTG	TGGCAGTCTCAGAATAGGCTTC
LAMC3	GCTCCGAGGAATGCACGTT	TGTCATCGCACTGGAGGTGTA
