# Supplementary material for: Targeting Oncogenic Wnt/β-Catenin Signaling in Adrenocortical Carcinoma Disrupts ECM Expression and Impairs Tumor Growth
Source: Cancers (Basel). 2023 Jul 10;15(14):3559. doi: 10.3390/cancers15143559 (PMC10377252; doi:10.3390/cancers15143559)
Supplement: Supplementary file 1 [file cancers-15-03559-s001.zip › Western Blots - File S1.pdf]

Full unedited gel imaged in two channels
Bands outlined in red are shown in Figure 3C

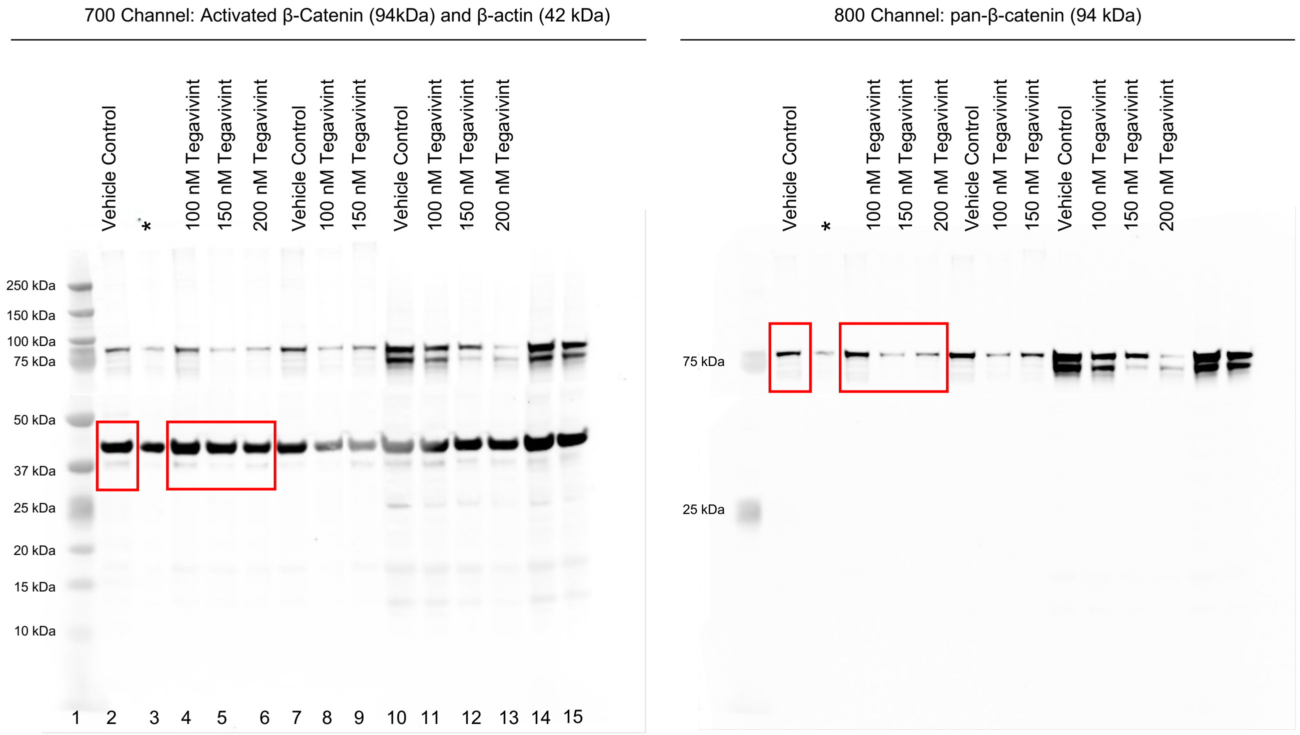

The membrane was probed with antibodies against pan-β-catenin (ThermoFisher Scientific MA1-2001, 1:1000), or β-actin (Sigma-Aldrich A-5441, 1:5000).

Rows 2-9 were extracted with RIPA; Rows 10-15 are biologic replicates extracted with a nuclear lysis protocol
* Row 3 was misloaded and therefore skipped

Full unedited gel imaged in two channels

Active β-catenin (94 kDa)

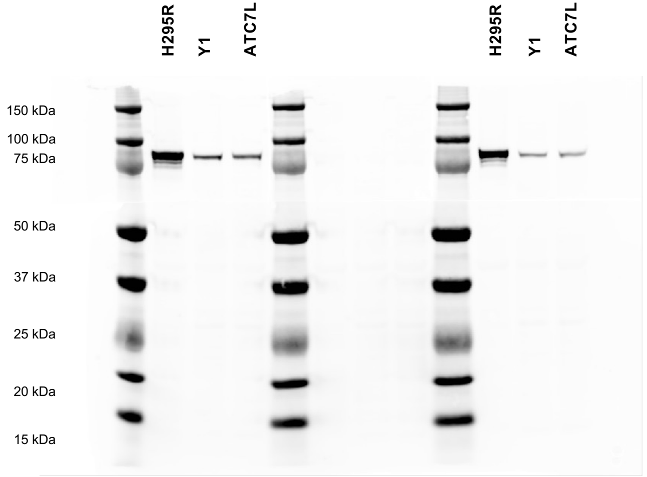

β-Catenin (94kDa) and β-actin (42 kDa)

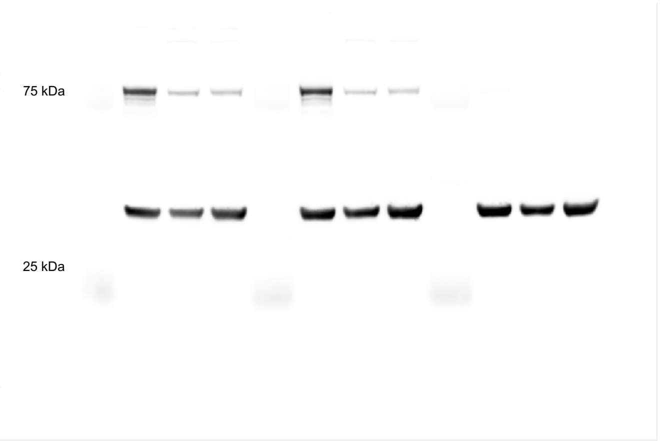

The left and right thirds of the membrane were probed with antibodies against Active β-Catenin (Cell Signaling #9561, 1:1500). The left and center thirds of the membrane were probed with antibodies against β-catenin (ThermoFisher Scientific MA1-2001, 1:1000). The bottom half of the membrane was probed with antibodies against β-actin (Sigma-Aldrich A-5441, 1:5000).

All protein lysates were extracted with RIPA
