# Supplementary figures and images for: Targeting Oncogenic Wnt/β-Catenin Signaling in Adrenocortical Carcinoma Disrupts ECM Expression and Impairs Tumor Growth
Source: Cancers (Basel). 2023 Jul 10;15(14):3559. doi: 10.3390/cancers15143559 (PMC10377252; doi:10.3390/cancers15143559)

NCI-H295R

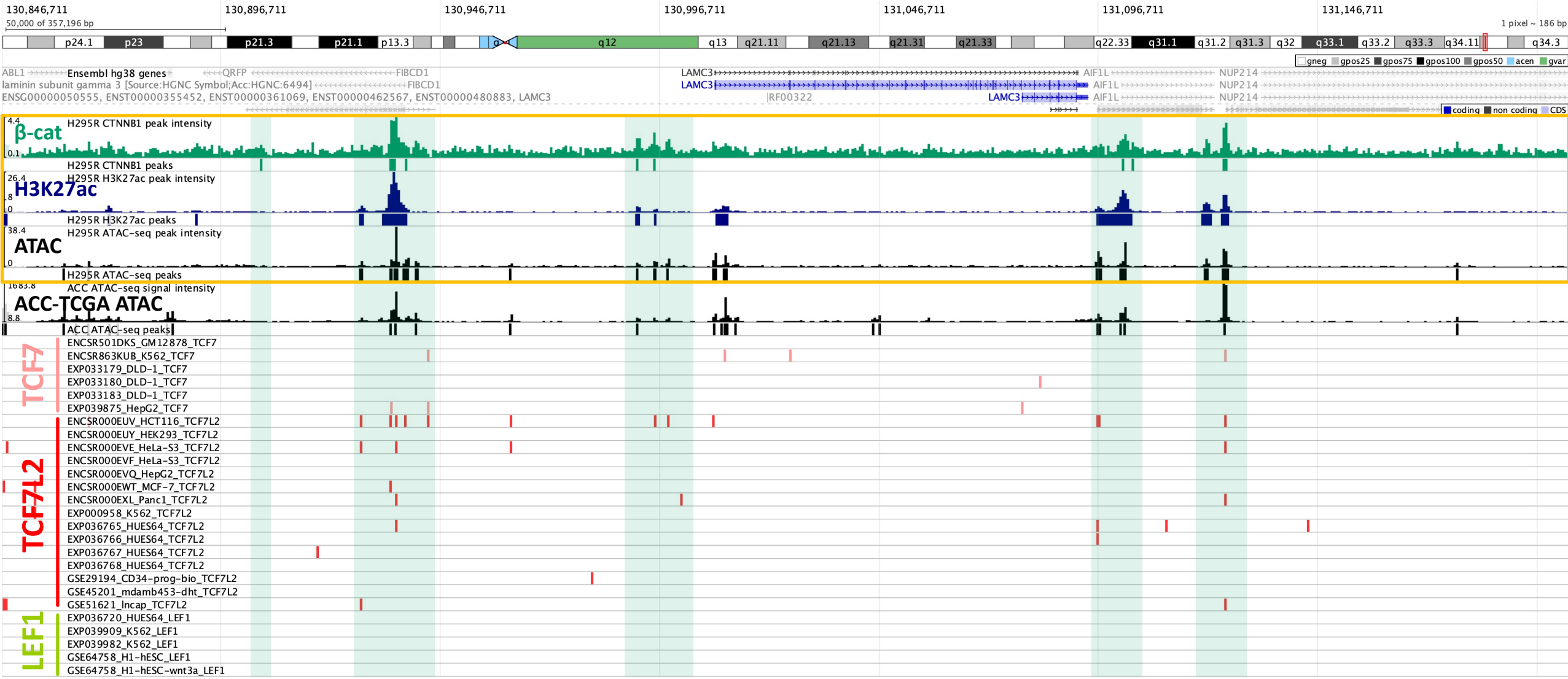

NCI-H295R

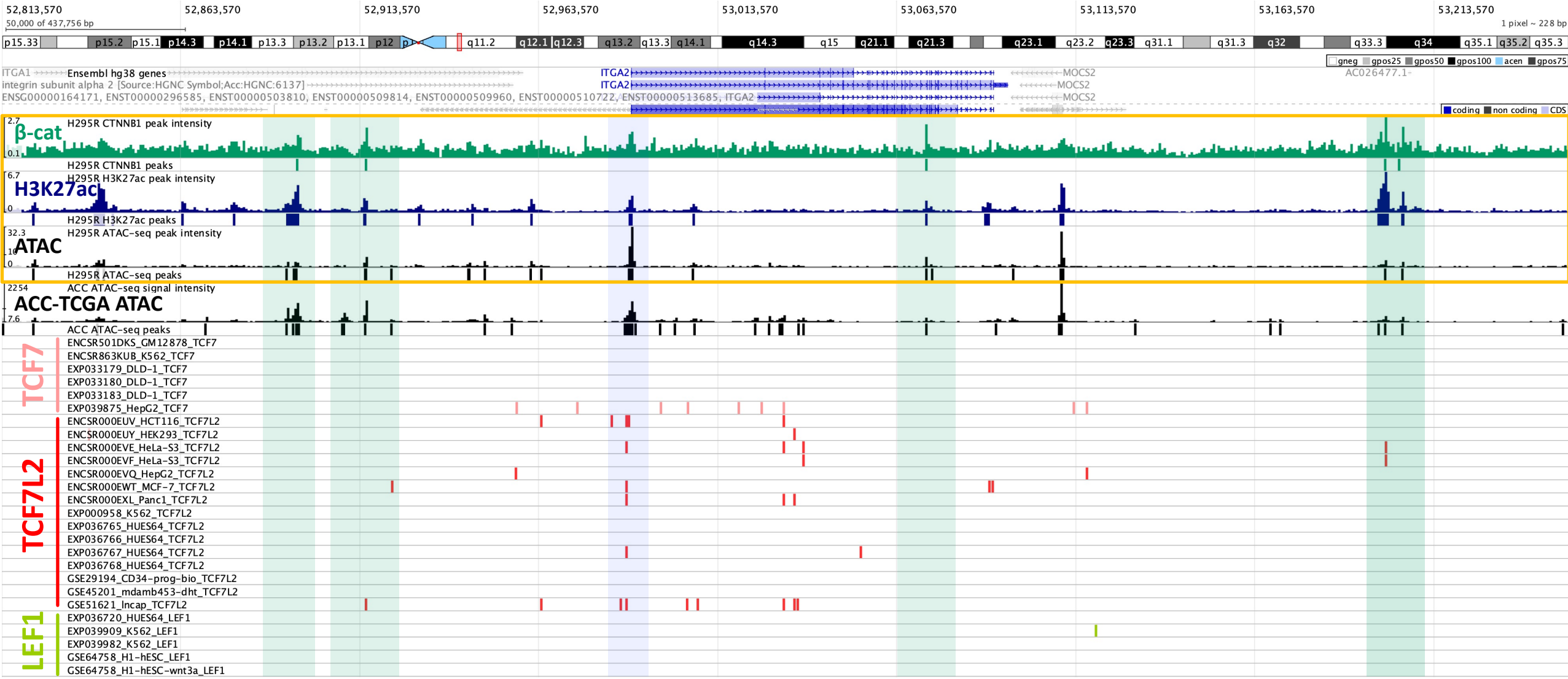

NCI-H295R

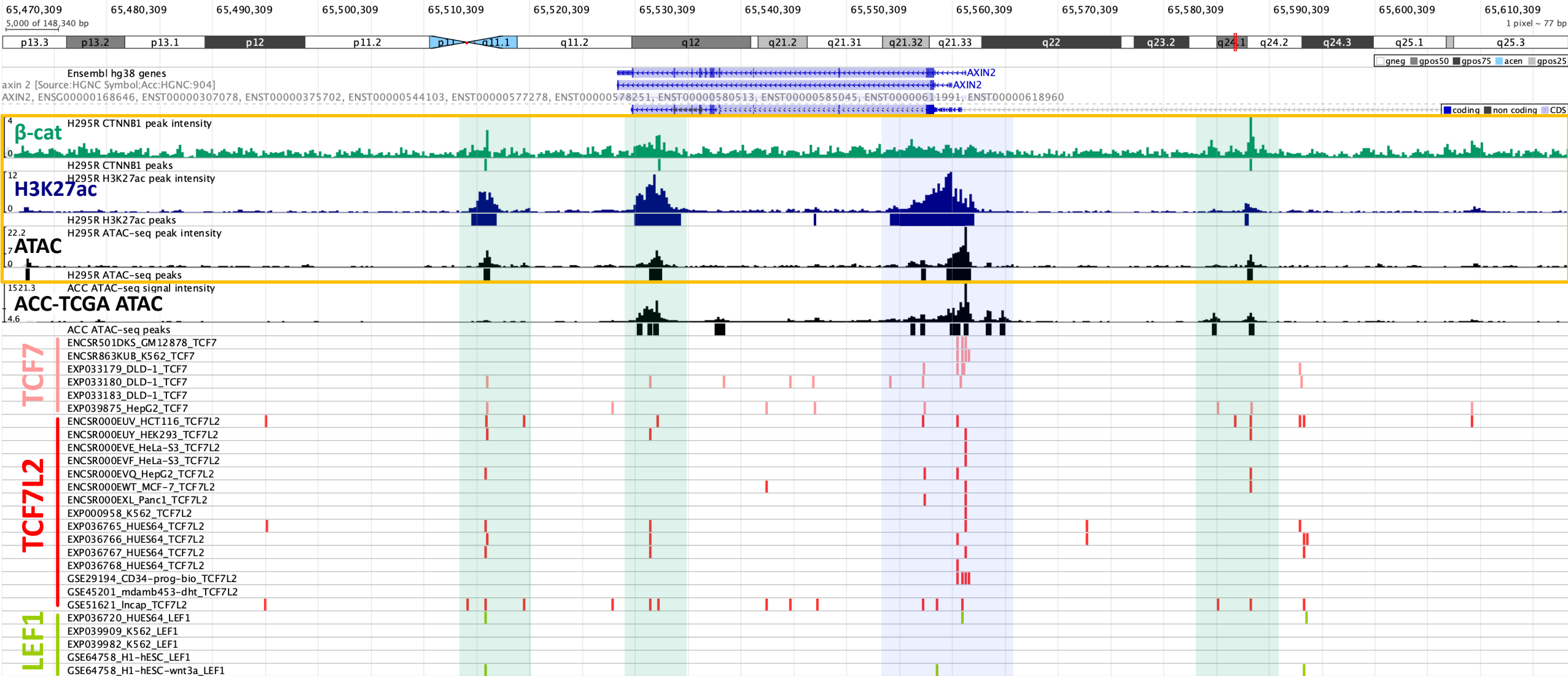

NCI-H295R

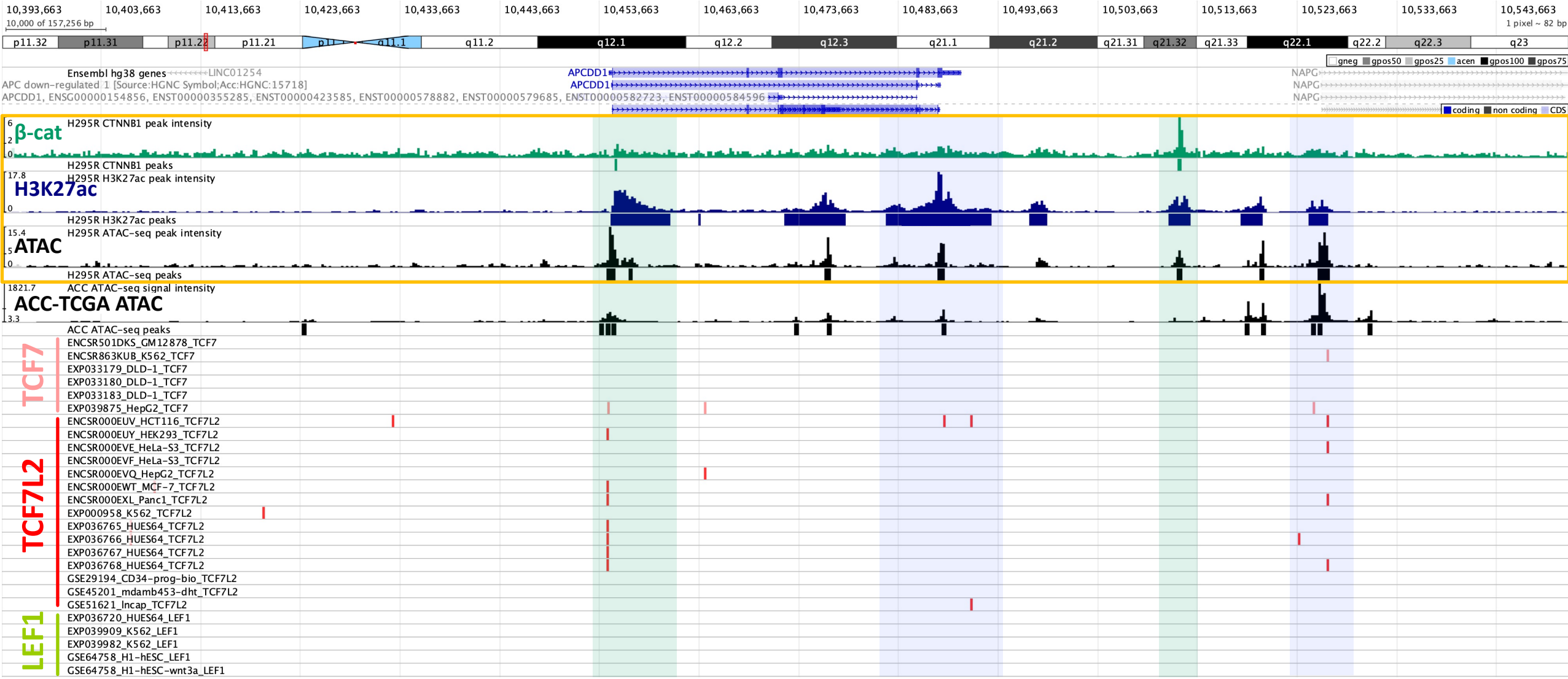

NCI-H295R

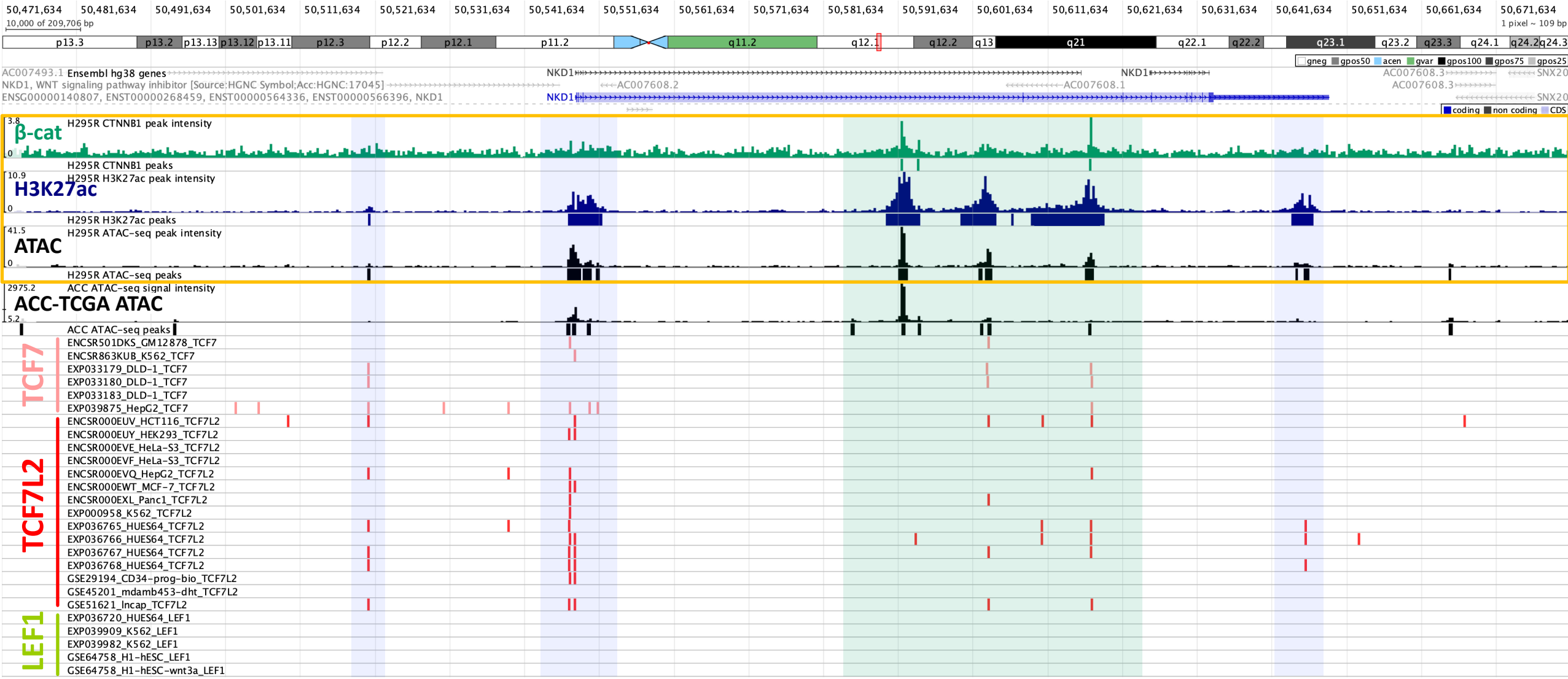

Supplement: Supplementary file 1 [file cancers-15-03559-s001.zip › Cancers Supplementary Figure S1 - Revisions.pdf]

A.

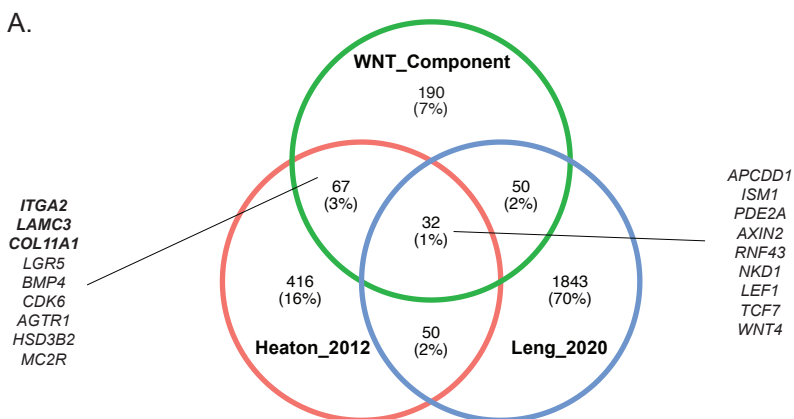

B.

## Overall Survival

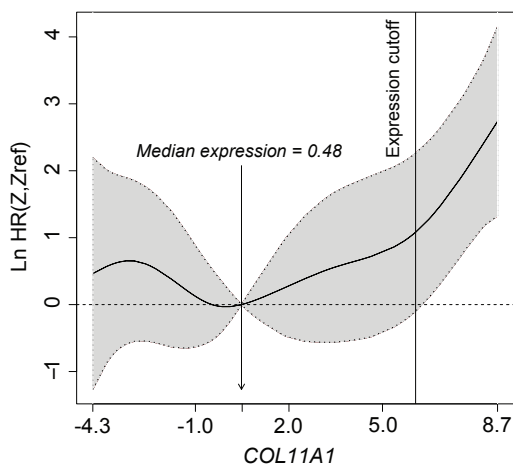

C.

## Disease Free Survival

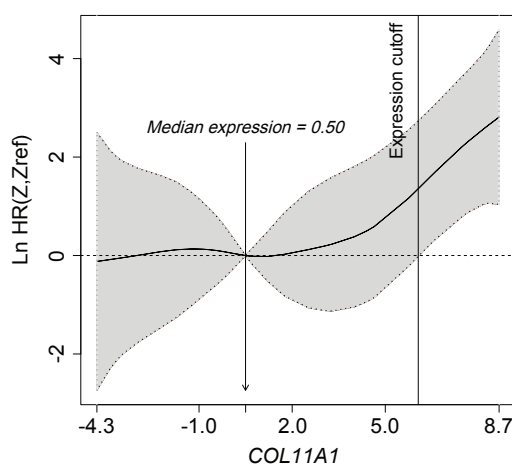

D.

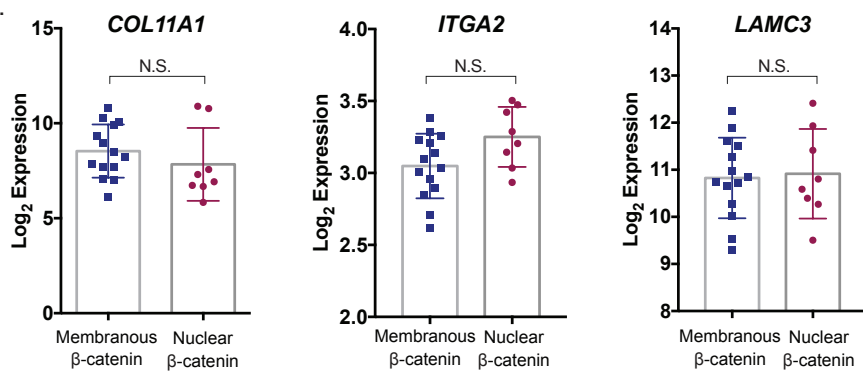

E.

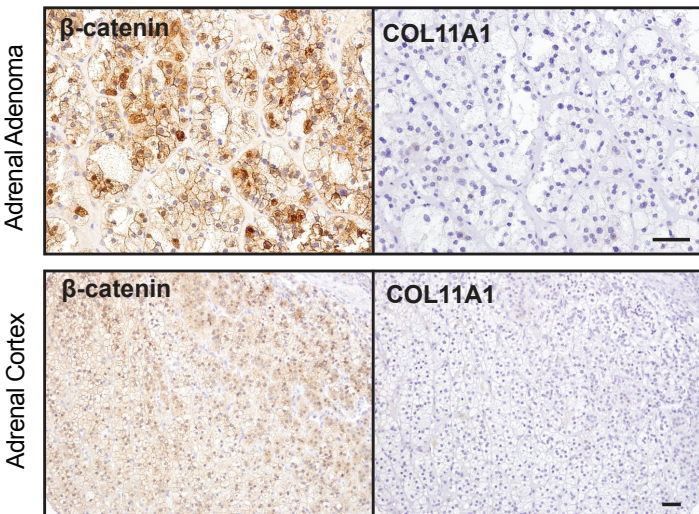

F.

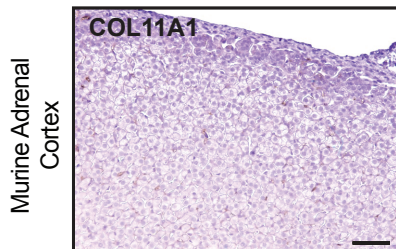

Supplement: Supplementary file 1 [file cancers-15-03559-s001.zip › Cancers Supplementary Figure S2 Revisions.pdf]

A.

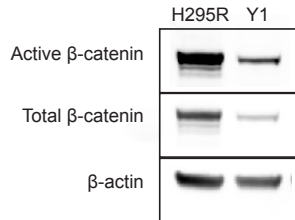

B.

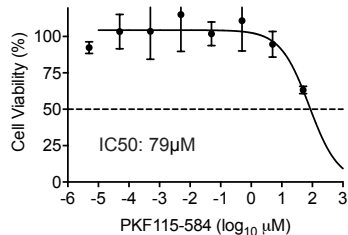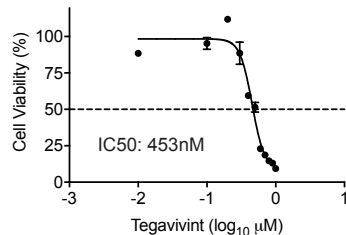

C.

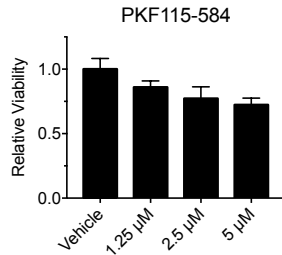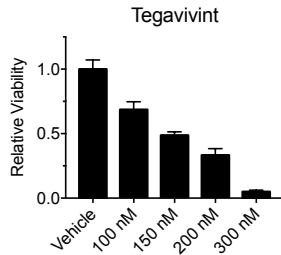

D.

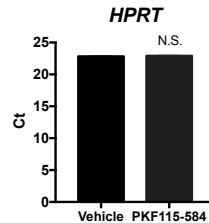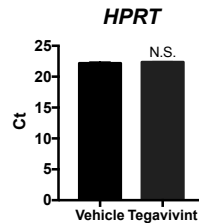

Supplement: Supplementary file 1 [file cancers-15-03559-s001.zip › Cancers Supplementary Figure S3 Revisions.pdf]

A.

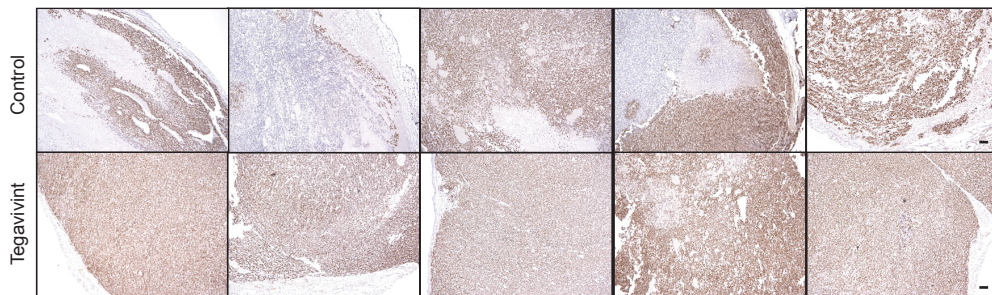

B.

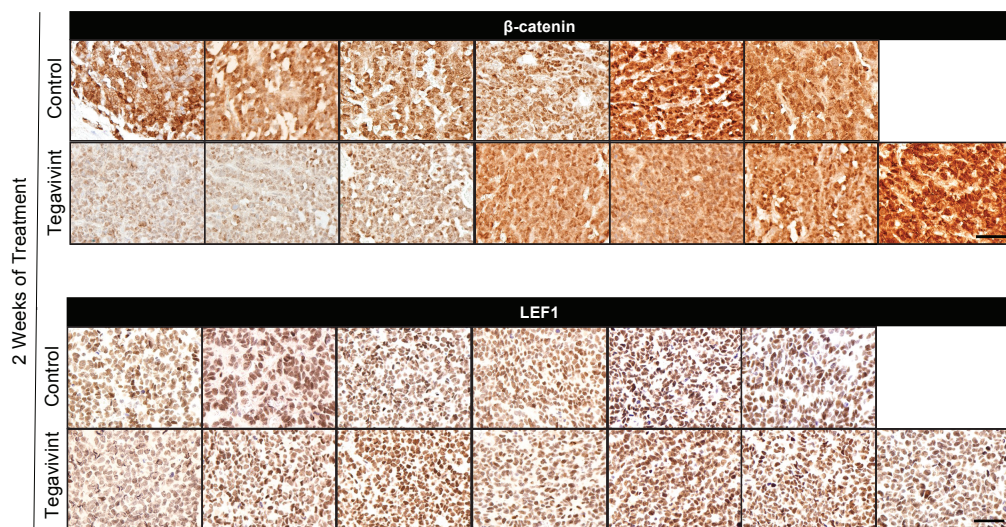

Supplement: Supplementary file 1 [file cancers-15-03559-s001.zip › Cancers Supplementary Figure S4 - Revisions.pdf]
